# Lithium for Bipolar Disorder and Risk of Thyroid Dysfunction and Chronic Kidney Disease

**DOI:** 10.1001/jamanetworkopen.2024.58608

**Published:** 2025-02-11

**Authors:** Joe Kwun Nam Chan, Marco Solmi, Christoph U. Correll, Corine Sau Man Wong, Heidi Ka Ying Lo, Francisco Tsz Tsun Lai, Wing Chung Chang

**Affiliations:** 1 Department of Psychiatry, Li Ka Shing Faculty of Medicine, The University of Hong Kong, Queen Mary Hospital, Hong Kong SAR, China; 2 SCIENCES Lab, Department of Psychiatry, University of Ottawa, Ottawa, Ontario, Canada; 3 Regional Centre for the Treatment of Eating Disorders, Department of Mental Health, The Ottawa Hospital, Ottawa, Ontario, Canada; 4 Clinical Epidemiology Program, Ottawa Hospital Research Institute, University of Ottawa, Ottawa, Ontario, Canada; 5 Department of Child and Adolescent Psychiatry, Charité Universitätsmedizin, Berlin, Germany; 6 On Track: The Champlain First Episode Psychosis Program, Department of Mental Health, The Ottawa Hospital, Ottawa, Ontario, Canada; 7 Department of Psychiatry, The Zucker Hillside Hospital, Northwell Health, Glen Oaks, New York; 8 Department of Psychiatry and Molecular Medicine, Donald and Barbara Zucker School of Medicine at Hofstra/Northwell, Hempstead, New York; 9 School of Public Health, Li Ka Shing Faculty of Medicine, The University of Hong Kong, Hong Kong SAR, China; 10 Centre for Safe Medication Practice and Research, Department of Pharmacology and Pharmacy, Li Ka Shing Faculty of Medicine, The University of Hong Kong, Hong Kong SAR, China; 11 Department of Family Medicine and Primary Care, School of Clinical Medicine, Li Ka Shing Faculty of Medicine, The University of Hong Kong, Hong Kong SAR, China; 12 Laboratory of Data Discovery for Health, Hong Kong Science Park, Sha Tin, Hong Kong SAR, China; 13 Advanced Data Analytics for Medical Science Limited, Hong Kong SAR, China; 14 State Key Laboratory of Brain and Cognitive Sciences, The University of Hong Kong, Hong Kong SAR, China

## Abstract

**Importance:**

Literature indicates adverse effects of lithium on thyroid and kidney function. However, existing data are heterogeneous, with limitations in quality and lack evaluation of adverse effects of lithium vs other mood stabilizers, especially commonly prescribed second-generation antipsychotics. Lithium serum level thresholds associated with thyroid and kidney abnormalities remain unknown.

**Objective:**

To examine risk of thyroid and kidney dysfunction in patients with incident bipolar disorder (BD) treated with lithium and other mood stabilizers and antipsychotics in an Asian population and to determine lithium serum level cutoffs associated with these physical complications.

**Design, Setting, and Participants:**

This population-based retrospective cohort study identified patients aged 15 years or older with first-diagnosed BD in Hong Kong from 2002 to 2018, utilizing a medical record database of public health care services. Data analysis was performed from February to May 2024.

**Exposures:**

Lithium vs nonlithium treatment. The nonlithium group was further stratified into valproate, olanzapine, quetiapine, and risperidone groups.

**Main Outcomes and Measures:**

Main outcomes were hypothyroidism, hyperthyroidism, and chronic kidney disease stage 3 or higher (CKD3+), with additional investigation on CKD stage 4 or higher (CKD4+) and end-stage kidney disease (ESKD). Outcomes were ascertained using laboratory test results. Cox proportional hazards regression analyses were performed for risk estimation with adjusted hazard ratios (aHRs) and 95% CIs. Receiver operating characteristic analyses with the Youden index were employed to determine lithium serum level cutoffs associated with thyroid and kidney dysfunction.

**Results:**

There were 4752 individuals with analyzable data for hypothyroidism (mean [SD] age, 39.5 [15.6] years; mean [SD] follow-up, 8.4 [4.8] years; 2889 female [60.8%]), 4500 with data for hyperthyroidism (mean [SD] age, 39.7 [15.6] years; mean [SD] follow-up, 8.7 [4.7] years; 2716 female [60.4%]), and 7029 with data for CKD (mean [SD] age, 37.9 [14.8] years; mean [SD] follow-up, 8.3 [4.8] years; 4251 female [60.5%]). Lithium was associated with increased risk of hypothyroidism (aHR, 2.00; 95% CI, 1.72-2.33) and CKD3+ (aHR, 1.35; 95% CI, 1.15-1.60), but not CKD4+ or ESKD, compared with nonlithium treatments. Higher lithium serum levels were associated with elevated rates of hypothyroidism (aHR, 2.08; 95% CI, 1.67-2.59), hyperthyroidism (aHR, 1.81; 95% CI, 1.31-2.50), and CKD3+ (aHR, 2.11; 95% CI, 1.57-2.85). Greater number of lithium toxicity episodes was associated with increased CKD3+ risk. Valproate, olanzapine, quetiapine, and risperidone generally exhibited reduced likelihood of thyroid dysfunction and CKD3+ compared with lithium, without any difference in advanced CKD. Mean lithium serum levels greater than 0.5028 mEq/L, greater than 0.5034 mEq/L, and greater than 0.5865 mEq/L represented thresholds associated with hypothyroidism, hyperthyroidism, and CKD3+, respectively.

**Conclusions and Relevance:**

In this cohort study of patients with incident BD, lithium was associated with a mildly increased risk of thyroid dysfunction and CKD in a predominantly Chinese population. The identified lithium level thresholds associated with risks of physical complications may facilitate the development of evidence-based guidelines recommending lithium treatment, particularly in Asian populations, and the promotion of personalized care and risk-benefit balancing in the treatment for BD.

## Key Points

**Question:**

Is lithium associated with increased risk of hypothyroidism, hyperthyroidism, and chronic-kidney-disease (CKD), and what thresholds of lithium serum levels are associated with thyroid and kidney dysfunction in people with bipolar disorder (BD)?

**Findings:**

In this cohort study of individuals with BD in Hong Kong, 4752 were analyzed hypothyroidism, 4500 for hyperthyroidism, and 7029 for CKD; lithium was associated with increased risk of hypothyroidism and CKD stage 3 or higher, but not more advanced CKD, compared with other mood stabilizers. Mean lithium serum levels greater than 0.5028 mEq/L, greater than 0.5034 mEq/L, and greater than 0.5865 mEq/L represented thresholds associated with hypothyroidism, hyperthyroidism, and CKD stage 3 or higher, respectively.

**Meaning:**

These data can provide important empirical evidence that can inform clinical guidelines on determining optimal range of lithium serum levels, balancing treatment efficacy and safety, and promoting personalized treatment for BD, particularly in Asian populations.

## Teaser

This cohort study analyzes associations of lithium treatment with hyperthyroidism, hypothyroidism, and chronic kidney disease among individuals with bipolar disorder in Hong Kong.

## Introduction

Lithium is a first-line maintenance pharmacotherapy for bipolar disorder (BD),^1^ effectively reducing recurrence, readmission, and self-harm.^2,3^ However, lithium has been underutilized,^4^ which is partly attributable to its narrow therapeutic index and reported increased risk of thyroid dysfunction^5,6^ and chronic kidney disease (CKD).^7^ Clinical guidelines recommend regular monitoring of thyroid and kidney functions in patients receiving lithium treatment for early detection and intervention.^1,8^ Nonetheless, evidence behind these recommendations is heterogeneous with limitations in quality. No study has assessed specific thresholds of lithium serum levels above which these adverse effects start to emerge.

Register-based studies have recently examined the risk of thyroid and kidney dysfunction associated with lithium treatment. A meta-analysis^7^ found an increased risk of impaired kidney function in patients treated with lithium, but with substantial cross-study heterogeneity. More recent reports have revealed less consistent findings^6,9,10,11^ and observed a lack of an association of lithium exposure with risk of CKD or decline in glomerular filtration rate.^6,9,10^ Previous research was also hampered by important limitations including modest sample size,^10,12,13,14^ short observation periods,^5,15^ disregarding the BD diagnosis^11,15,16^ (independently associated with increased risk of CKD),^17^ inadequate confounder adjustment without considering physical comorbidities, prescriptions of other psychotropics and nephrotoxic medications,^10,11,13,16^ focusing on prevalent BD samples,^5^ and lack of outcome ascertainment for advanced CKD, which is less subject to surveillance bias.^6,9,10,11,12,13,15^ Few studies have systematically investigated the associations of thyroid and kidney dysfunction with lithium treatment characteristics, encompassing lithium serum levels, cumulative treatment duration, and toxicity episodes.^5,6,13^ Data are lacking regarding specific cutoffs for lithium serum levels predicting these adverse effects, limiting the determination of optimal therapeutic range that balances treatment efficacy and safety. Second-generation antipsychotics (SGAs) are increasingly prescribed as mood stabilizers for BD.^4^ However, the risk of thyroid and kidney dysfunction associated with SGAs and mood-stabilizing anticonvulsants is understudied. Data on the association of lithium with impaired thyroid and kidney functioning is primarily derived from Western countries, which might not generalize to the global East due to substantial cross-regional variations in ethnicities, health care systems, and prescribing practices.

This population-based cohort study aimed to examine the risk of hypothyroidism, hyperthyroidism, and CKD associated with lithium treatment among patients with an initial diagnosis of BD over a 17-year period in Hong-Kong, utilizing an electronic health record (EHR) database of public health care services. Associations of lithium treatment characteristics with adverse outcomes were evaluated, particularly optimal thresholds of lithium serum levels associated with thyroid dysfunction and CKD. We also assessed the comparative risk of adverse outcomes for alternative mood stabilizers of valproate and 3 of the most commonly prescribed SGAs (quetiapine, olanzapine, and risperidone) with reference to lithium.

## Methods

### Data Source and Study Population

This population-based cohort study was approved by the institutional review board of the University of Hong Kong and Hospital Authority Hong Kong–West cluster and followed the Strengthening the Reporting of Observational Studies in Epidemiology (STROBE) and Reporting of Studies Conducted Using Observational Routinely Collected Data (RECORD) reporting guidelines. Data for this study were extracted from the Clinical Data Analysis and Reporting System (CDARS), an EHR database developed by the Hospital Authority, a statutory body delivering government subsidized, universal health coverage to all Hong Kong residents by managing all public hospitals and specialist and general outpatient clinics in Hong Kong.^18^ CDARS is an integrated, longitudinal patient-record system capturing clinical and prescribing data across all health care settings of Hospital Authority facilities. We identified all individuals who received their first-ever diagnosis of BD (*International Statistical Classification of Diseases and Related Health Problems, Tenth Revision [ICD-10]* codes F30-F31) for public psychiatric inpatient or outpatient care and were aged 15 years or older at diagnosis between January 1, 2002, and December 31, 2018, as the study cohort Details about the data source and study population are in the eMethods in Supplement 1. Follow-up of patients began on the date of first-recorded BD diagnosis until the occurrence of the specified outcome, death, or December 31, 2018, whichever came first. Because individual patient records in this database were completely unidentifiable, no informed consent was required.

### Study Outcomes, Exposure, and Covariates of Interest

Hypothyroidism, hyperthyroidism, and CKD stage 3 or higher (CKD3+) represented primary study outcomes. Hypothyroidism and hyperthyroidism were ascertained by the first-recorded thyrotropin level greater than 5 mIU/L and less than 0.35 mIU/L at follow-up. CKD3+ was defined as having 2 measurements of estimated glomerular filtration rate (eGFR; calculated using the CKD Epidemiology Collaboration equation)^19^ less than 60 mL/min/1.73 m^2^ separated by 3 or more months at follow-up. More advanced CKD outcomes, including CKD stage 4 or higher (CKD4+; eGFR <30 mL/min/1.73 m^2^), and end-stage kidney disease (ESKD; eGFR <15 mL/min/1.73 m^2^) with and without kidney replacement (dialysis or transplantation) therapy, were also assessed.

Incidence of thyroid and kidney outcomes was compared between patients with and without lithium treatment. Lithium exposure was defined as filling 1 or more prescriptions of lithium during study follow-up. Information of lithium treatment characteristics, including number of lithium blood tests, mean serum levels, cumulative duration, and number of toxicity episodes during follow-up, were examined. Patients without lithium use (nonlithium group) served as the comparison group. To evaluate the comparative risk for adverse thyroid and kidney outcomes of alternative mood stabilizers relative to lithium, we further categorized patients in the nonlithium group into valproate, olanzapine, quetiapine, and risperidone users. Patients who concomitantly or sequentially filled 1 or more prescriptions of 1 or more of these 4 mood stabilizers were classified as users of the medication with the longest cumulative exposure duration during follow-up. In this set of analyses, patients without prescription records of lithium or these 4 mood stabilizers were excluded. A comprehensive array of covariates was included in the analyses, comprising patient demographics, pre-existing physical comorbidities, substance and alcohol use disorders, use of other psychotropics, and use of medications with nephrotoxic risk when interacting with lithium. Details of covariates are in the eMethods in Supplement 1).

### Statistical Analysis

We performed Cox proportional hazards regression analyses to examine the risk of hypothyroidism, hyperthyroidism, and CKD in patients treated with lithium, with reference to the nonlithium group, with baseline variables that were significantly different between the 2 groups being used as covariates. Subgroup analyses stratified by sex and age (<40, 40-59, and ≥60 years) were also conducted. Five sets of sensitivity analyses were performed to verify robustness of the results, including restricting analyses to patients treated with (1) a medication-possession ratio of 80% or greater to ensure good adherence and the predominance of exposure to lithium, (2) lithium as the patient’s first-ever mood stabilizer during the study period, (3) cumulative lithium exposure duration of 30 days or more to minimize misclassification bias, (4) 2 or more measurements of lithium serum levels, and (5) the mean lithium serum level greater than the median of the lithium serum levels of the entire lithium group. We repeated Cox regression models within the lithium group to assess the associations of risk of thyroid and kidney outcomes with lithium treatment characteristics, including mean lithium serum levels, cumulative treatment duration, and number of toxicity episodes (>1.0 mEq/L, >1.2 mEq/L, and >1.5 mEq/L [to convert to millimoles per liter, multiply by 1]). These lithium treatment characteristics were median-split for analyses, with patients having values below the median as the reference. We then conducted the time-dependent receiver operating characteristic (ROC) analyses to calculate area under the curve (AUC), taking into consideration the person-year exposure in Cox proportional hazards regression models, and Youden index (sensitivity + [1 – specificity]) was calculated to determine the optimal cutoffs of mean lithium serum levels associated with thyroid dysfunction and CKD3+ in patients treated with lithium.

Additionally, we performed Cox regression models to evaluate comparative risk of hypothyroidism, hyperthyroidism, CKD3+, and ESKD associated with valproate, quetiapine, olanzapine, and risperidone use, with the lithium group as the reference. Two sets of sensitivity analyses were conducted. First, only patients with a medication-possession ratio of 80% or greater for the specified mood stabilizer were included in the analyses to ensure good medication adherence. Second, a monotherapy analysis was conducted by restricting analyses to patients prescribed the specified mood stabilizer within the entire study period only in the respective exposure group to avoid confounding effects of other studied mood stabilizers. Results of all Cox regression models were presented as hazard ratios (HRs) with 95% CIs. The Bonferroni correction for multiple comparisons was applied to all of the study outcome analyses. eFigure 1 in Supplement 1 depicts a flowchart summarizing the primary (lithium vs nonlithium groups) and additional analyses (comparisons between lithium and alternative mood stabilizers), and their subgroup and sensitivity analyses, while eTable 1 in Supplement 1 provides descriptions about the study populations, exposure, and covariates for each of the subgroups, and sensitivity analyses. All statistical analyses were performed using R version 4.1.2. (R Project for Statistical Computing), with 2-sided testing and a significance level of α < .05. Analyses were conducted from February to May 2024.

## Results

Altogether, 4752 patients (mean [SD] age, 39.5 [15.6] years; mean [SD] follow-up, 8.4 [4.8] years; 2889 female [60.8%]; 1725 treated with lithium [36.3%]) were analyzed for hypothyroidism, 4500 (mean [SD] age, 39.5 [15.6] years; mean [SD] follow-up, 8.7 [4.7] years; 2716 female [60.4%]; 1538 treated with lithium [34.2%]) for hyperthyroidism, and 7029 (mean [SD] age, 37.9 [14.8] years; mean [SD] follow-up, 8.3 [4.8] years; 4251 female [60.5%]; 2258 treated with lithium [32.1%]) for CKD outcomes. More than 85% of lithium users had 2 or more measurements of lithium serum levels (eFigure 2 in Supplement 1), with the mean (SD) number of lithium blood tests ranging between 8.4 (8.3) and 9.8 (10.1) (Table 1). The mean (SD) lithium serum level was 0.5 (0.2) mEq/L for all 3 study outcomes; mean (SD) cumulative duration ranged from 3.6 (4.1) years to 4.2 (4.3) years (Table 1).

**Table 1.** Baseline Characteristics of Patients With Bipolar Disorder Treated With vs Without Lithium in the Analysis for Thyroid and Kidney Outcomes

Characteristics^a^	Participants, No. (%)								
	Hypothyroidism (N = 4752)			Hyperthyroidism (N = 4500)			Chronic kidney disease (N = 7029)		
	Lithium (n = 1725)	No lithium (n = 3027)	*P *value	Lithium (n = 1538)	No lithium (n = 2962)	*P* value	Lithium (n = 2258)	No lithium (n = 4771)	*P* value
Age, mean (SD), y	34.4 (13.1)	40.1 (16.5)	<.001	34.4 (13.0)	40.1 (16.4)	<.001	34.1 (12.9)	39.7 (15.2)	<.001
Sex									
Female	979 (56.8)	1910 (63.1)	<.001	851 (55.3)	1865 (63.0)	<.001	1328 (58.8)	2923 (61.3)	.05
Male	746 (43.2)	1117 (36.9)		687 (44.7)	1097 (37.0)		930 (41.2)	1848 (38.7)	
Medical comorbidities^b^									
Hypertension	44 (2.6)	245 (8.1)	<.001	35 (2.3)	238 (8.0)	<.001	48 (2.1)	303 (6.4)	<.001
Dyslipidemia	22 (1.3)	108 (3.6)	<.001	20 (1.3)	108 (3.6)	<.001	25 (1.1)	178 (3.7)	<.001
Diabetes	21 (1.2)	131 (4.3)	<.001	19 (1.2)	139 (4.7)	<.001	22 (1.0)	181 (3.8)	<.001
Alcohol use disorder	12 (0.7)	24 (0.8)	.71	10 (0.7)	25 (0.8)	.48	14 (0.6)	39 (0.8)	.34
Substance use disorder	20 (1.2)	46 (1.5)	.31	22 (1.4)	51 (1.7)	.46	25 (1.1)	76 (1.6)	.09
Charlson Comorbidity Index score, mean (SD)^c^	0.6 (0.9)	1.0 (1.5)	<.001	0.6 (0.9)	1.0 (1.5)	<.001	0.5 (0.8)	0.9 (1.3)	<.001
Lithium treatment characteristics									
No. of lithium blood tests, mean (SD)	8.4 (8.3)	NA	NA	9.0 (8.9)	NA	NA	9.8 (10.1)	NA	NA
Lithium serum level, mean (SD), mEq/L^d^	0.5 (0.2)	NA	NA	0.5 (0.2)	NA	NA	0.5 (0.2)	NA	NA
Duration of lithium exposure, mean (SD), y	3.6 (4.1)	NA	NA	4.1 (4.3)	NA	NA	4.2 (4.3)	NA	NA
No. of lithium toxicity episodes, mean (SD)^e^	0.4 (1.1)	NA	NA	0.4 (1.2)	NA	NA	0.5 (1.2)	NA	NA
Use of other psychotropics									
Antipsychotics	1645 (95.4)	2744 (90.7)	<.001	1458 (94.8)	2680 (90.5)	<.001	2145 (95.0)	4180 (87.6)	<.001
Anticonvulsants^f^	1261 (73.1)	2379 (78.6)	<.001	1108 (72.0)	2269 (76.6)	.001	1676 (74.2)	3586 (75.3)	.34
Antidepressants	1015 (58.8)	1868 (61.7)	.051	933 (60.7)	1820 (61.4)	.61	1345 (59.6)	2736 (57.3)	.08
Use of other medications									
NSAIDs	NA	NA	NA	NA	NA	NA	430 (19.0)	977 (20.5)	.16
ACEIs or ARBs	NA	NA	NA	NA	NA	NA	180 (8.0)	565 (11.8)	<.001
Diuretics	NA	NA	NA	NA	NA	NA	95 (4.2)	306 (6.4)	<.001
Catchment areas^g^									
Hong Kong East	121 (7.0)	196 (6.5)	.09	102 (6.6)	194 (6.5)	.09	153 (6.8)	289 (6.1)	.002
Hong Kong West	116 (6.7)	171 (5.6)		111 (7.2)	173 (5.8)		169 (7.5)	295 (6.2)	
Kowloon Central	213 (12.3)	410 (13.5)		212 (13.8)	423 (14.3)		282 (12.5)	680 (14.3)	
Kowloon East	256 (14.8)	407 (13.4)		236 (15.3)	379 (12.8)		347 (15.4)	615 (12.9)	
Kowloon West	226 (13.1)	460 (15.2)		216 (14.0)	458 (15.5)		298 (13.2)	758 (15.9)	
New Territories East	511 (29.6)	896 (29.6)		419 (27.2)	836 (28.2)		634 (28.1)	1327 (27.8)	
New Territories West	281 (16.3)	478 (15.8)		240 (15.6)	488 (16.5)		364 (16.1)	780 (16.3)	

Abbreviations: ACEIs, angiotensin-converting enzyme inhibitors; ARBs, angiotensin receptor blockers; NA, not applicable; NSAIDs, nonsteroidal anti-inflammatory drugs.

^a^ χ^2^ tests and independent samples *t* tests were conducted for analysis of categorical and continuous variables, respectively.

^b^ Medical comorbidity represented the presence of the stated comorbidities at baseline.

^c^ Charlson Comorbidity score calculation excluded diabetes items and represented the comorbidity severity at baseline.

^d^ To convert to millimoles per liter, multiply by 1.

^e^ Lithium toxicity is defined as lithium serum level greater than 1.0 mEq/L.

^f^ Anticonvulsants referred to any prescriptions of valproate, carbamazepine, or lamotrigine.

^g^ In Hong Kong, the Hospital Authority manages public health care service delivery (inpatient and specialist or general outpatient services) which is mainly organized into 7 clusters based on geographical locations (ie, catchment areas).

**Figure 1.**
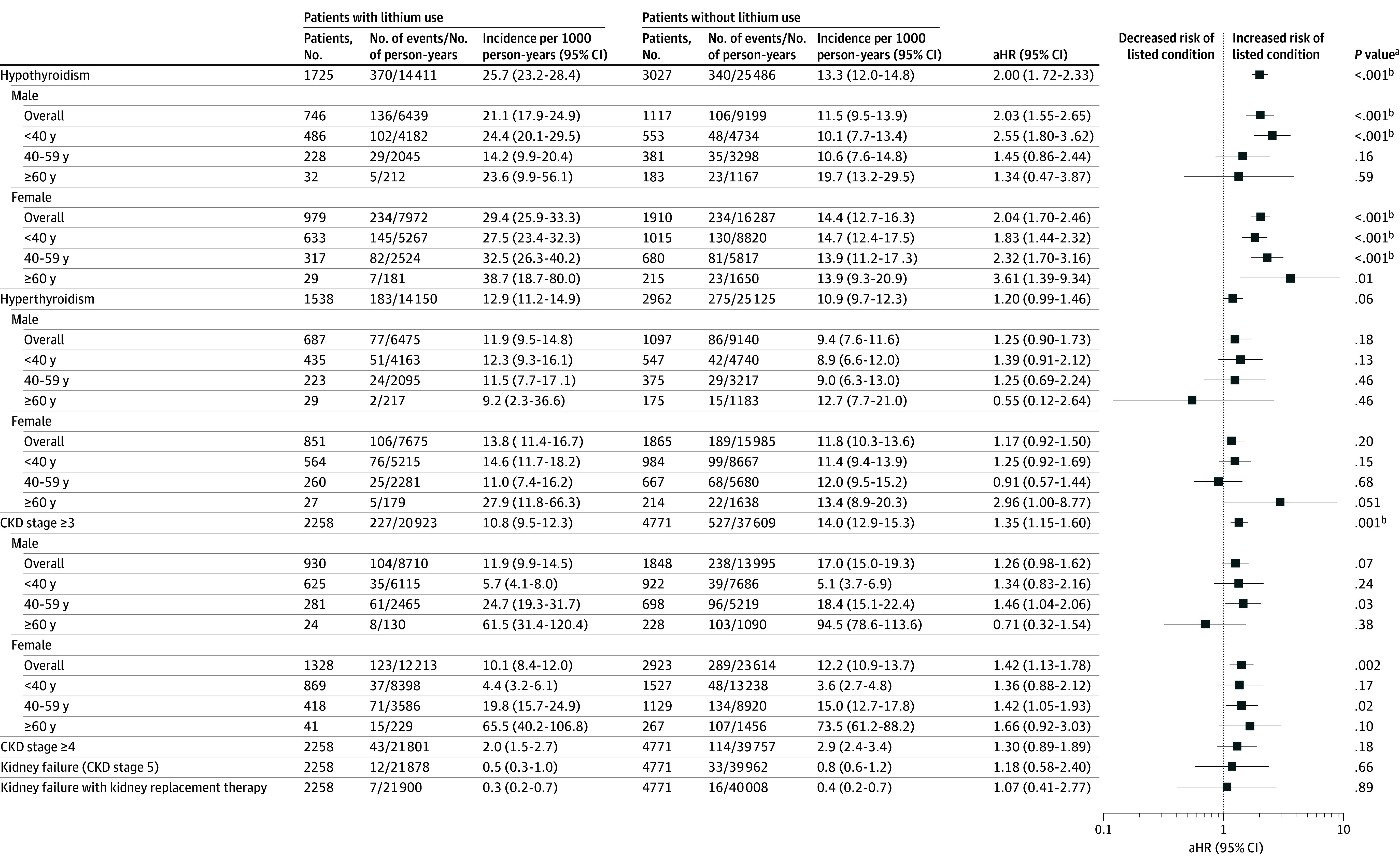
Lithium Use and Risk of Hypothyroidism, Hyperthyroidism, and Chronic Kidney Disease (CKD) in Patients With Bipolar Disorder aHR indicates adjusted hazard ratio. ^a^Bonferroni correction for multiple comparison was applied (corrected *P* value = .002). ^b^Results remained significant after Bonferroni correction.

In lithium users, the incidence rates per 1000 person-years were 25.7 (95% CI, 23.2-28.4) for hypothyroidism, 12.9 (95% CI, 11.2-14.9) for hyperthyroidism, and 10.8 (95% CI, 9.5-12.3) for CKD3+ (Figure 1). Lithium users had significantly higher risk of hypothyroidism (adjusted HR [aHR], 2.00; 95% CI, 1.72-2.33) and CKD3+ (aHR, 1.35; 95% CI, 1.15-1.60) than the nonlithium group (Figure 1). No between-group difference was observed in the risk of hyperthyroidism, CKD4+, and ESKD with or without kidney replacement. Kaplan-Meier curves depicting cumulative incidence of thyroid and CKD3+ outcomes are shown in eFigure 3 in Supplement 1. In sex- and age-stratified analyses, lithium was associated with elevated risk of hypothyroidism in both sexes and all age categories except for males aged 40 to 59 years and 60 years or older, as well as an increased risk of CKD3+ in females and males aged 40 to 59 years. Results of sensitivity analyses were generally consistent with the primary analyses (eTable 2 and eTable 3 in Supplement 1). Although lithium was associated with increased risk of hyperthyroidism in the sensitivity analyses when restricting the sample to lithium users with lithium as their first-used mood stabilizer (aHR, 1.38; 95% CI, 1.03-1.85) and with mean lithium serum levels greater than median serum levels of the entire lithium group (aHR, 1.38; 95% CI, 1.09-1.74), these associations became nonsignificant after Bonferroni correction.

**Figure 2.**
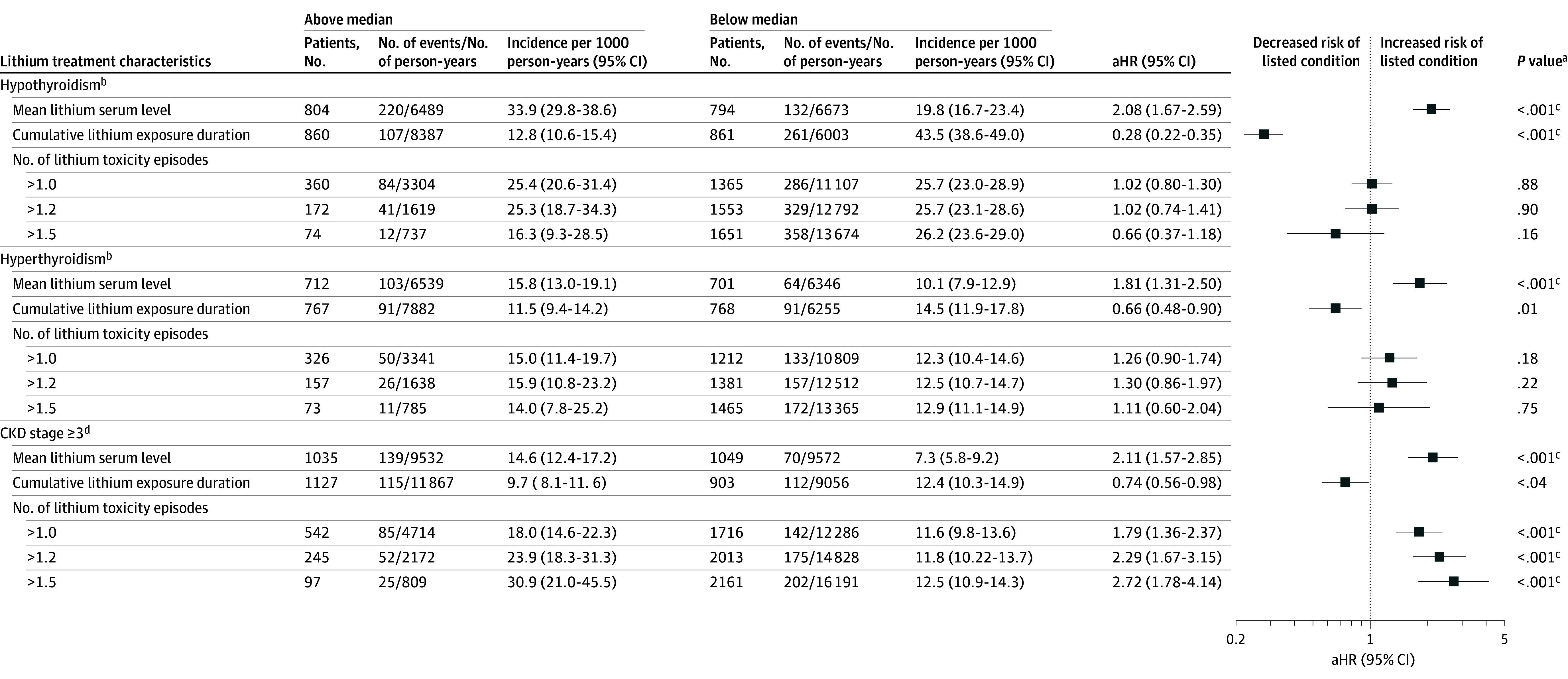
Lithium Treatment Characteristics and Risk of Hypothyroidism, Hyperthyroidism, and Chronic Kidney Disease (CKD) in Patients With Bipolar Disorder The median value of mean lithium serum level of lithium users included for hypothyroidism, hyperthyroidism, and CKD outcomes were 0.51 mEq/L, 0.50 mEq/L, and 0.52 mEq/L, respectively (to convert to millimoles per liter, multiply by 1). The median value of cumulative lithium exposure duration of lithium users included for hypothyroidism, hyperthyroidism, and CKD outcomes were 1.95 years, 2.45 years, and 2.47 years, respectively. The median value of number of lithium toxicity episodes for the 3 study outcomes (>1.0 mEq/L, >1.2 mEq/L, and >1.5 mEq/L) were all 0. aHR indicates adjusted hazard ratio. ^a^Bonferroni correction was applied for multiple comparison (threshold for *P* value = .003). ^b^Regression models adjusted for age at bipolar disorder diagnosis, sex, hypertension, dyslipidemia, diabetes, age-adjusted Charlson Comorbidity Index score, and prescriptions of other antipsychotics (ie, antipsychotics other than olanzapine, quetiapine, and risperidone), other mood-stabilizing anticonvulsants (ie, carbamazepine or lamotrigine), any antidepressants, and studied mood stabilizers other than the specified agent. ^c^Results remained significant after Bonferroni correction. ^d^Regression models adjusted for age at bipolar disorder diagnosis, sex, hypertension, hyperlipidemia, diabetes, age-adjusted Charlson Comorbidity Index score, catchment area of psychiatric service receipt, and prescriptions of other antipsychotics, mood-stabilizing anticonvulsants, antidepressant, nonsteroid anti-inflammatory drugs, angiotensin converting enzyme inhibitors or angiotensin II receptor blocks, and diuretics.

**Figure 3.**
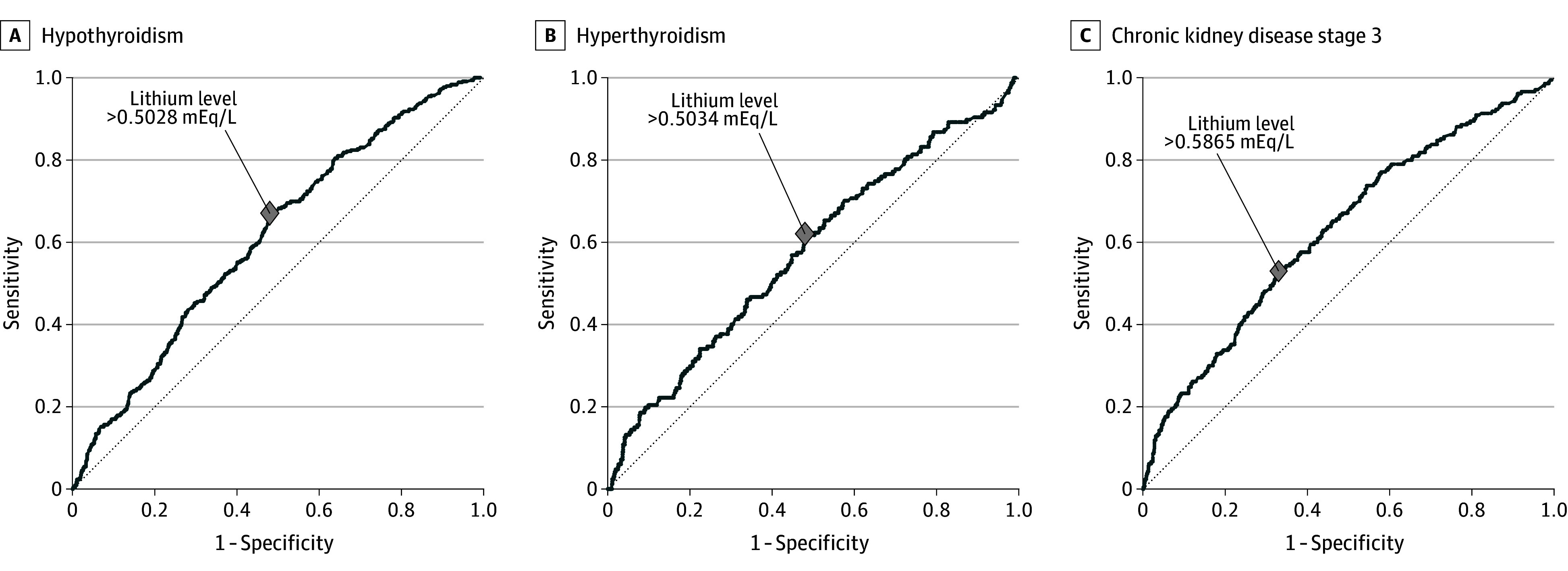
Receiver Operating Characteristic Analysis on Mean Lithium Serum Levels Associated With Occurrence of Adverse Outcomes Mean lithium serum levels greater than 0.5028 mEq/L (sensitivity = 0.67; specificity = 0.52), greater than 0.5034 mEq/L (sensitivity = 0.62; specificity = 0.52), and greater than 0.5865 mEq/L (sensitivity = 0.53; specificity = 0.67) was associated with occurrence of hypothyroidism (A), hyperthyroidism (B), and chronic kidney disease stage 3 (C), respectively. To convert to millimoles per liter, multiply by 1.

Concerning lithium treatment characteristics, higher lithium serum levels were associated with an increased risk of hypothyroidism (aHR, 2.08; 95% CI, 1.67-2.59), hyperthyroidism (aHR, 1.81; 95% CI, 1.31-2.50), and CKD3+ (aHR, 2.11; 95% CI, 1.57-2.85) (Figure 2). A longer cumulative lithium treatment duration was associated with lower risk of hypothyroidism (aHR, 0.28; 95% CI, 0.20-0.35), hyperthyroidism (aHR, 0.66; 95% CI, 0.48-0.90), and CKD3+ (aHR, 0.74; 95% CI, 0.56-0.98). Nonetheless, only the association of lithium treatment duration with hypothyroidism remained significant after Bonferroni correction. A greater number of toxicity episodes, irrespective of lithium serum level cutoffs, was associated with elevated risk of CKD3+ but not thyroid dysfunction. The ROC analyses showed that the mean lithium serum level was significantly associated with hypothyroidism (AUC, 0.61; 95% CI, 0.58-0.64; *P* < .001), hyperthyroidism (AUC, 0.58; 95% CI, 0.53-0.63; *P* < .001), and CKD3+ (AUC, 0.63; 95% CI, 0.59-0.67; *P* < .001), with the Youden indices of greater than 0.5028 mEq/L for hypothyroidism, greater than 0.5034 mEq/L for hyperthyroidism, and greater than 0.5865 mEq/L for CKD3+ (Figure 3).

Compared with lithium use, use of valproate (aHR, 0.47; 95% CI, 0.39-0.56), quetiapine (aHR, 0.47; 95% CI, 0.36-0.62), olanzapine (aHR, 0.30; 95% CI, 0.19-0.48), and risperidone (aHR, 0.51; 95% CI, 0.36-0.73) were associated with reduced risk of hypothyroidism (Table 2). Valproate users had lower risk of hyperthyroidism (aHR, 0.51; 95% CI, 0.40-0.66) and CKD3+ (aHR, 0.75; 95% CI, 0.61-0.92) relative to patients treated with lithium, while olanzapine users displayed decreased risk of hyperthyroidism (aHR, 0.29; 95% CI, 0.14-0.58) and quetiapine users displayed decreased risk of CKD3+ (aHR, 0.65; 95% CI, 0.49-0.86). Of note, the association of valproate with CKD3+ became nonsignificant after Bonferroni correction. Valproate and each SGA did not differ from lithium regarding ESKD risk. Sensitivity analyses generally yielded similar results (eTable 4 in Supplement 1).

**Table 2.** Risk of Hypothyroidism, Hyperthyroidism, and CKD in Patients With Bipolar Disorder Treated With Lithium and Other Mood Stabilizers^a^

Condition	No. of Events/total No. of participants	Person-years, No.	Incidence rate per 1000 person-years (95% CI)	Adjusted HR (95% CI)	*P* value^b^
Hypothyroidism (n = 4479)^c^					
Lithium	370/1725	14 411	25.7 (23.2-28.4)	1 [Reference]	NA
Valproate	179/1549	13 854	12.9 (11.2-14.9)	0.47 (0.39-0.56)	<.001^d^
Olanzapine	18/272	2064	8.7 (5.5-13.8)	0.30 (0.19-0.48)	<.001^d^
Quetiapine	64/652	4598	13.9 (10.9-17.8)	0.47 (0.36-0.62)	<.001^d^
Risperidone	35/281	2408	14.5 (10.5-20.2)	0.51 (0.36-0.73)	<.001^d^
Hyperthyroidism (n = 4199)^c^					
Lithium	183/1538	14 150	12.9 (11.2-14.9)	1 [Reference]	NA
Valproate	105/1475	13 505	7.8 (6.4-9.4)	0.51 (0.40-0.66)	<.001^d^
Olanzapine	8/262	1994	4.0 (2.0-8.0)	0.29 (0.14-0.58)	.001^d^
Quetiapine	52/640	4510	11.5 (8.8-15.1)	0.74 (0.54-1.04)	.08
Risperidone	38/284	2444	15.5 (11.3-21.3)	1.04 (0.73-1.50)	.83
CKD stage 3 or higher (n = 6503)^e^					
Lithium	227/2258	20 923	10.8 (9.5-12.3)	1 [Reference]	NA
Valproate	244/2457	20 814	11.7 (10.3-13.3)	0.75 (0.61-0.92)	.006
Olanzapine	21/378	2544	8.3 (5.4-12.6)	0.64 (0.41-1.01)	.06
Quetiapine	77/1004	6601	11.7 (9.3-14.6)	0.65 (0.49-0.86)	.002^d^
Risperidone	39/406	3307	12.0 (8.6-16.1)	0.71 (0.50-1.02)	.06
End-stage kidney disease (n = 6503)^e^^,^^f^					
Lithium	12/2258	20 923	0.6 (0.3-1.0)	1 [Reference]	NA
Valproate	13/2457	21 843	0.6 (0.3-1.0)	0.88 (0.19-4.05)	.87
Olanzapine	2/378	2601	0.8 (0.2-3.1)	1.85 (0.25-13.44)	.55
Quetiapine	7/1004	6830	1.0 (0.5-2.1)	1.24 (0.24-6.38)	.80
Risperidone	2/406	3467	0.6 (0.1-2.3)	0.61 (0.08-4.60)	.63

Abbreviations: CKD, chronic kidney disease; HR, hazard ratio.

^a^ Patients with bipolar disorder without any lithium use were regarded as users of 1 of the 4 other mood stabilizers according to their prescription records. If patients were concomitantly or sequentially exposed to more than 1 alternative mood stabilizer, they were regarded as users of the medication group with the longest cumulative exposure duration.

^b^ Bonferroni correction for multiple comparisons was applied (corrected *P* value = .003).

^c^ Regression models were adjusted for age at bipolar disorder diagnosis, sex, catchment area of psychiatric service receipt, hypertension, dyslipidemia, diabetes, alcohol and substance use disorders, age-adjusted Charlson Comorbidity Index score, as well as prescription records of other antipsychotics (ie, antipsychotics other than olanzapine, quetiapine, or risperidone), other mood-stabilizing anticonvulsants (ie, carbamazepine or lamotrigine), any antidepressants, and studied mood stabilizers other than the specified agent.

^d^ Results remained significant after Bonferroni correction.

^e^ Regression models were adjusted for age at bipolar disorder diagnosis, sex, catchment area of psychiatric service receipt, hypertension, dyslipidemia, diabetes, alcohol and substance use disorders, age-adjusted Charlson Comorbidity Index score, and prescription of other antipsychotics, other mood-stabilizing anticonvulsants, any antidepressants, and studied mood stabilizers other than the specified agent, nonsteroid anti-inflammatory drugs, angiotensin-converting enzyme inhibitors or angiotensin II receptor blockers, and diuretics.

^f^ End-stage kidney disease referred to CKD stage 5 with or without kidney replacement therapy.

## Discussion

In this population-based cohort study of patients with first-diagnosed BD over a 17-year observation period, we affirmed the results of prior research demonstrating an elevated risk of hypothyroidism associated with lithium treatment vs valproate and commonly used SGAs.^6,11,20^ Although the primary analysis showed that lithium was not associated with an increased risk of hyperthyroidism, the association became significant in sensitivity analyses restricting the analyses to lithium users with high lithium serum levels and those prescribed lithium as the first-ever mood stabilizer (albeit those associations did not survive multiple-comparison correction). Our findings concur, at least partially, with a systematic review^21^ and a recent study^6^ indicating hyperthyroidism as a lithium-related adverse effect despite its comparatively lower prevalence rate. Intriguingly, consistent with an earlier, large-scale UK study,^11^ our results showed that shorter lithium exposure duration was associated with increased risk of hypothyroidism and hyperthyroidism, suggesting that thyroid dysfunction might occur early after initiation of lithium treatment. A recent study^22^ further observed that most thyroid dysfunction (approximately 90%) manifested within 3 years of commencing lithium, while another report^14^ found that 60% of patients treated with lithium who developed thyroid abnormalities did so within 5 years of treatment. Importantly, however, a considerable proportion of patients with BD who receive lithium therapy do not receive guideline-concordant laboratory monitoring as part of usual care.^9^ Our data thus underscore the importance of adhering to the guideline recommendations to facilitate early detection and intervention of lithium-induced thyroid dysfunction.

Consistent with a recent meta-analysis,^7^ we observed that lithium use was associated with an increased risk of developing CKD3+. Our data showed a 1.35-fold higher risk of CKD3+ in lithium users than nonlithium users, which is slightly lower than the 1.5- to 2-fold increased risk generally reported in prior research.^7,23^ The attenuated CKD risk estimate in our study might partly be attributable to the minimized confounding due to our adjustment for a more comprehensive array of covariates than used previously, encompassing physical comorbidities, substance and alcohol use disorders, and other psychotropics and nephrotoxic medications, which were not adequately controlled for in most previous studies. In line with recent studies including advanced stages of CKD as adverse outcomes,^17,24^ our results revealed no association of lithium use with CKD4+ and ESKD, being thereby contrary to the findings of earlier research reporting increased occurrence of ESKD in patients treated with lithium in the 1960s to 1980s^16^ when higher lithium serum levels (0.8-1.2 mEq/L) were recommended for maintenance therapy of BD. Several possible reasons might explain the discrepant findings. The lower lithium levels and regular kidney function monitoring recommended by recent clinical guidelines^1,8,25^ might have substantially reduced the risk for ESKD. However, it is also possible that the relatively short cumulative lithium exposure duration in our lithium-treated sample of approximately 8.5 years might be too brief to capture advanced CKD, given that evidence suggests a prolonged latency period (up to 10-20 years) for the occurrence of ESKD in patients receiving long-term lithium therapy.^25^ The small event number of ESKD can also compromise the statistical power to detect group differences. Nevertheless, accumulating data revealed that an earlier stage of kidney impairment in patients treated with lithium may not progress to advanced CKD or ESKD. A recent review of 4 studies assessing the risk of CKD progression in lithium users noted that all of these studies found no increased ESKD risk with lithium continuation vs discontinuation.^26^ Conversely, evidence has consistently shown that patients with BD who discontinue lithium exhibit significantly higher risk of mood-episode recurrence than those who continue lithium.^27^ Hence, our findings indicate that lithium-related CKD risk should be weighed carefully against the efficacy in mood-episode recurrence prevention and antisuicidal properties of lithium therapy in BD.

We found that higher lithium serum levels and greater number of toxicity episodes were associated with an increased risk of CKD3+, supporting the dose-response association of lithium treatment with kidney impairment.^11,23,28^ Importantly, this is the first study, to our knowledge, to specifically determine the cutoffs of lithium levels associated with thyroid and kidney dysfunction in BD. Our ROC analyses showed that these adverse outcomes were associated with serum levels of 0.50 to 0.58 mEq/L, which are lower than the therapeutic range of 0.60 to 0.80 mEq/L recommended by some clinical guidelines for lithium maintenance treatment.^1,8^ Notably, the median lithium levels of our samples ranged 0.50 to 0.52 mEq/L. In fact, the precise therapeutic range for lithium maintenance treatment of BD remains unclear. A recent meta-analysis and a study of Chinese patients with BD^29^ found that lithium serum levels of 0.40-0.80 mEq/L significantly prevented mood episode recurrence, while a systematic review^25^ indicated 0.40 mEq/L as the minimally-effective lithium level for maintenance treatment. There has been evidence, albeit limited, suggesting that ethnicity may be associated with treatment response to lithium in BD, with some earlier reports showing that Asian individuals may respond to lithium at lower serum levels than individuals of other ethnicities.^30^ Our results thus indicate the critical importance to determine the minimum effective dose for lithium to attain therapeutic effect and mitigate potential adverse effects. Owing to the paucity of data, further investigation examining the optimal thresholds of lithium levels in predicting thyroid and kidney dysfunction, taking into account the potential moderating effect of ethnicity, is warranted to inform clinical guidelines for lithium treatment. Alternatively, similar to a previous UK study,^11^ we observed a negative association of lithium treatment duration with the risk of CKD3+. This finding is contrary to a recent meta-analysis^7^ showing that a longer duration of lithium exposure was associated with an increased likelihood of CKD, although the association with CKD risk disappeared in post hoc meta-regression analyses when studies examining elderly patient samples were excluded. Our finding might plausibly be attributable to surveillance bias, which otherwise unlikely affects advanced CKD outcomes, and to potential reverse causality due to early lithium discontinuation based on detection of abnormal kidney function. Some studies also reported a greater decline in eGFR among patients with longer lithium treatment duration.^31,32,33^ These findings, however, may not be directly comparable to ours because the current investigation focused on the occurrence of specific stages of CKD rather than eGFR trajectories, which may not necessarily reach the diagnostic threshold of CKD outcomes. Of note, the result that lithium treatment duration was associated with an increased risk of CKD3+ did not survive multiple-comparison correction. Future research is required to clarify whether a subgroup of patients with BD may be at high risk of developing kidney impairment early in the course of lithium treatment.

The current study is among the few in the world and the first, to our knowledge, in Asia to assess the comparative risk of thyroid and kidney dysfunction between lithium and other major mood stabilizers, including valproate and frequently used SGAs. We observed that valproate, olanzapine, quetiapine, and risperidone users had a lower risk of hypothyroidism than lithium users. Our analyses also revealed that valproate and olanzapine were associated with a reduced risk of hyperthyroidism, while valproate and quetiapine were associated with decreased CKD3+ risk, relative to lithium (although the association of valproate with CKD3+ became nonsignificant after multiple-comparison correction). No difference was found in the risk of CKD4+ or ESKD between lithium and any of these 4 alternative mood stabilizers. Limited population-based studies revealed similar results, showing a decreased risk of thyroid dysfunction in patients treated with valproate and olanzapine compared with lithium.^5,34^ Mixed findings were observed across those few studies regarding the comparative risk of kidney impairment. A UK study^5^ reported a lower risk of CKD3+ in patients with BD treated with valproate, olanzapine, and quetiapine than those treated with lithium, while a Canadian study^15^ observed decreased risk of 30% or greater loss in eGFR in valproate users compared with lithium users. However, 2 recent studies^9,10^ demonstrated lack of significant differences between lithium and valproate groups in annual eGFR decline during follow-up, whereas an earlier Danish study^17^ showed that the use of mood-stabilizing anticonvulsants, but not lithium or antipsychotics, was associated with an increased ESKD risk. Given the scarcity of existing data, further research is needed to systematically evaluate the comparative safety profiles of lithium and other mood stabilizers for BD.

### Strengths and Limitations

This study has several strengths. We examined a large population-based cohort of patients with incident BD over a long observation period. Important confounders, including physical comorbidities and medications associated with nephrotoxicity, were adjusted for in the analyses. Comparative risk of thyroid and kidney dysfunction of alternative mood stabilizers, including valproate and 3 of the most commonly prescribed SGAs with reference to lithium, were evaluated. Availability of lithium serum level data enabled evaluation of the associations of lithium treatment characteristics with observed adverse outcomes. In particular, we applied ROC analyses to identify thresholds of lithium levels associated with thyroid abnormalities and CKD. Our study data, which were based on a regionally generalizable sample of patients with BD in a public health care system in Hong Kong, can be specifically useful in facilitating formulation of recommendations on lithium treatment in Asian populations.

There are also several limitations in the study. First, the observational nature of this study precluded random assignment of medication treatment and preplanned assessments regarding frequency of follow-up and laboratory testing. However, our EHR-derived, routinely collected clinical, medication, and laboratory data reflected the prescribing practices and outcomes observed in clinical settings and were therefore more representative and generalizable that the data obtained from the randomized clinical trials. Moreover, EHR-based research enables patient cohorts with a large sample size to be assessed over a long follow-up period to facilitate a more adequate capture of rare outcomes for study analyses such as CKD or ERKD. Second, data on lifestyle variables such as physical activity, dietary patterns, smoking, and body mass index were not adequately recorded in the medical database and thus were not adjusted for in the analyses. Third, similar to most other pharmacoepidemiological studies, patients’ exposure to prescribed mood-stabilizing medications was derived from dispensing records, which may overestimate the actual intake of medications in the study cohort. Fourth, ascertainment of exposure to lithium and other mood stabilizers for the outcome analyses was based on the cumulative medication treatment duration within the study follow-up (due to lack of information on the exact prescription dates for time-dependent Cox models), which might be subject to immortal time bias. Our sensitivity analyses with a medication-possession ratio of 80% or greater for mood stabilizers, which partially addressed this limitation and ensured the predominance of exposure to the specified mood stabilizers within the study follow-up period, produced generally consistent results as the primary analyses and supported the robustness of the study results. It is noteworthy that this limitation might also affect subgroup and other sensitivity analyses, and future investigations are required to verify the respective findings. Fifth, the study data did not contain information denoting disorder subtypes, clinical polarity, and symptom severity, precluding us from exploring the moderation effect of specific illness-related features of BD on the association of lithium use with the risk of thyroid and kidney dysfunction. Sixth, medication dose of nonlithium mood stabilizers was not available, and hence the dose-response association of valproate and the studied SGAs with adverse outcomes could not be evaluated. Seventh, lithium level monitoring occurred as part of usual care, with varying number and frequencies, affecting mean lithium serum level calculations for the outcome analyses. Eighth, we focused on the risk of lithium and alternative mood stabilizers for thyroid and kidney pathology and did not analyze treatment benefits to put these data into a risk-benefit context, which was beyond the scope of this study. Nevertheless, despite these limitations, the current study provides new and relevant information on the frequency, relative risk, and correlates of thyroid and kidney adverse effects of lithium treatment in patients with incident BD, coming from a complete public health care service database in Hong Kong that can inform clinical decision making and patient care.

## Conclusions

In this territory-wide EHR-based cohort study of patients with first-diagnosed BD in a predominantly Chinese population, lithium use was associated with a modestly increased risk of thyroid dysfunction and CKD stage 3, but not more advanced CKD or ESKD, relative to treatment with alternative mood stabilizers including valproate and commonly prescribed SGAs. The risk of these adverse outcomes was accentuated with exposure to higher lithium levels and lithium intoxication, particularly CKD. Our findings further identified thresholds of lithium serum levels associated with thyroid and kidney abnormalities, thereby providing empirical evidence to inform clinical guidelines on recommending lithium treatment that balances the efficacy and safety; this in turn can facilitate promotion of personalized care and equity in the treatment of BD in Asian populations. Future investigation systematically determining the optimal therapeutic range of lithium maintenance treatment for prevention of BD recurrence is warranted. Development of risk-prediction algorithms for CKD in patients with BD treated with lithium would further facilitate individualized psychopharmacotherapy in clinical practice. Clinicians should balance efficacy and the effect of lithium in delaying mortality in those with BD with safety risk and concerns^35^ in a shared decision-making process with each patient.

## Notes

Supplement 1.
eMethods.
**eFigure 1.** Flowchart Showing Primary, Additional, Subgroup and Sensitivity Analyses for the Risk of Hypothyroidism, Hyperthyroidism and Chronic Kidney Disease in Bipolar Disorder Patients **eTable 1.** Description of Study Population, Exposure, Covariates and Outcome in Subgroup and Sensitivity Analyses **eFigure 2.** (A) Number of Lithium Blood Tests of Lithium Users, and (B) Duration of Cumulative Exposure in Lithium Users in the Study Samples for (i) Hypothyroidism, (ii) Hyperthyroidism, and (iii) Chronic Kidney Disease **eFigure 3.** Cumulative Hazard for (A) Hypothyroidism, (B) Hyperthyroidism and (C) Chronic Kidney Disease in Bipolar Disorder Patients With Versus Without Lithium Use **eTable 2.** Sensitivity Analyses on Association Between Lithium Use and Risk of Hypothyroidism, Hyperthyroidism and Chronic Kidney Diseases in Bipolar Disorder Patients **eTable 3.** Association Between Lithium Use as First-Ever Mood-Stabilizer and Risk of Hypothyroidism, Hyperthyroidism and Chronic Kidney Diseases in Bipolar Disorder Patients **eTable 4.** Sensitivity Analyses on Risk of Hypothyroidism, Hyperthyroidism and Chronic Kidney Diseases in Bipolar Disorder Patients With Lithium and Other Mood-Stabilizers

Supplement 2.
Data Sharing Statement
