# Supplementary material for: Lithium for Bipolar Disorder and Risk of Thyroid Dysfunction and Chronic Kidney Disease
Source: JAMA Netw Open. 2025 Feb 11;8(2):e2458608. doi: 10.1001/jamanetworkopen.2024.58608 (PMC11815528; doi:10.1001/jamanetworkopen.2024.58608)
Supplement: Supplement 1. — eMethods. eFigure 1. Flowchart Showing Primary, Additional, Subgroup and Sensitivity Analyses for the Risk of Hypothyroidism, Hyperthyroidism and Chronic Kidney Disease in Bipolar Disorder Patients eTable 1. Description of Study Population, Exposure, Covariates and Outcome in Subgroup and Sensitivity Analyses eFigure 2. (A) Number of Lithium Blood Tests of Lithium Users, and (B) Duration of Cumulative Exposure in Lithium Users in the Study Samples for (i) Hypothyroidism, (ii) Hyperthyroidism, and (iii) Chronic Kidney Disease eFigure 3. Cumulative Hazard for (A) Hypothyroidism, (B) Hyperthyroidism and (C) Chronic Kidney Disease in Bipolar Disorder Patients With Versus Without Lithium Use eTable 2. Sensitivity Analyses on Association Between Lithium Use and Risk of Hypothyroidism, Hyperthyroidism and Chronic Kidney Diseases in Bipolar Disorder Patients eTable 3. Association Between Lithium Use as First-Ever Mood-Stabilizer and Risk of Hypothyroidism, Hyperthyroidism and Chronic Kidney Diseases in Bipolar Disorder Patients eTable 4. Sensitivity Analyses on Risk of Hypothyroidism, Hyperthyroidism and Chronic Kidney Diseases in Bipolar Disorder Patients With Lithium and Other Mood-Stabilizers [file jamanetwopen-e2458608-s001.pdf]

Supplemental Online Content

Chan JKN, Solmi M, Correll CU, et al. Risk of thyroid dysfunction and chronic kidney disease associated with lithium for bipolar disorder. *JAMA Netw. Open*. 2025;8(2):e2458608. doi:10.1001/jamanetworkopen.2024.58608

eMethods.

eFigure 1. Flowchart Showing Primary, Additional, Subgroup and Sensitivity Analyses for the Risk of Hypothyroidism, Hyperthyroidism and Chronic Kidney Disease in Bipolar Disorder Patients

eTable 1. Description of Study Population, Exposure, Covariates and Outcome in Subgroup and Sensitivity Analyses

eFigure 2. (A) Number of Lithium Blood Tests of Lithium Users, and (B) Duration of Cumulative Exposure in Lithium Users in the Study Samples for (i) Hypothyroidism, (ii) Hyperthyroidism, and (iii) Chronic Kidney Disease

eFigure 3. Cumulative Hazard for (A) Hypothyroidism, (B) Hyperthyroidism and (C) Chronic Kidney Disease in Bipolar Disorder Patients With Versus Without Lithium Use

eTable 2. Sensitivity Analyses on Association Between Lithium Use and Risk of Hypothyroidism, Hyperthyroidism and Chronic Kidney Diseases in Bipolar Disorder Patients

eTable 3. Association Between Lithium Use as First-Ever Mood-Stabilizer and Risk of Hypothyroidism, Hyperthyroidism and Chronic Kidney Diseases in Bipolar Disorder Patients

eTable 4. Sensitivity Analyses on Risk of Hypothyroidism, Hyperthyroidism and Chronic Kidney Diseases in Bipolar Disorder Patients With Lithium and Other Mood-Stabilizers

© 2025 Chan JKN et al. *JAMA Network Open*.

eMethods

Data source

The study data were extracted from the Clinical Data Analysis and Reporting System (CDARS), a territory-wide electronic health-record (EHR) database developed by the Hospital Authority (HA) which is a statutory body delivering government-subsidized, universal health coverage to all HK residents (a population of approximately 7.5 million in HK, with 92% being Chinese) by managing all public hospitals, specialist and general outpatient clinics in HK. CDARS is an integrated, longitudinal patient EHR system capturing clinical data across all healthcare settings of HA facilities. The database contains patients' demographics and clinical information including diagnoses, attendances to outpatient clinics and emergency departments, hospital admissions, laboratory tests, and prescribing/dispensing records of medications. Data on dates and causes of death were retrieved from CDARS via internal linkage to regional death registries from the Immigration Department. Patients' death status was also directly recorded and verified by CDARS as the vast majority of deaths in HK occur in public hospitals, thereby facilitating accurate ascertainment of death. Clinical data are collected and entered into computerized clinical-management system (CMS) by treating clinicians and other healthcare professionals, and are then transferred to CDARS for audit and research purposes. CDARS generates unique, anonymized patient identifiers to protect privacy and to link all medical records. This database has been used to conduct high-quality population-based studies on mental disorders including schizophrenia and BD,[1-7] and pharmaco-epidemiological investigations of psychotropic medications.[8-16] This study followed the STROBE and RECORD reporting guidelines,[17] and was approved by the Institutional Review board of the University of Hong-Kong/HA HK-West Cluster (UW 18-655). Since individual patient records in this database were completely unidentifiable, no informed consent was required.

Study population

We identified all individuals who received their first-ever diagnosis of BD (International Classification of Disease, 10th revision [ICD-10] codes F30–F31) for public psychiatric inpatient admission or outpatient care, and aged ≥15 years at diagnosis within 1-January-2002 and 31-December-2018 as the study population. Follow-up of patients began on the date of first-recorded BD diagnosis, until the occurrence of the specified outcome, death or 31-December-2018, whichever came first. Patients with BD who had their principal diagnosis changed to schizophrenia or schizoaffective disorder (ICD-10 F20 and F25) during study follow-up were excluded. In the analyses for thyroid outcomes (i.e. hypothyroidism and hyperthyroidism), patients were excluded if they had less than two records of thyroid stimulating hormone (TSH) tests, or had a recorded diagnosis of any thyroid disease (ICD-9-CM codes: 193, 242–246), receipt of any thyroxine treatment and/or TSH level <0·35 mIU/L or >5 mIU/L before the study follow-up or at baseline. In the analyses for renal outcomes (i.e. chronic kidney disease, CKD), patients were excluded if they had less than two recorded measurements of serum creatinine, or had recorded diagnosis of any renal disease (ICD-9-CM codes: 580–587, 589– 591, 593·2, 593·7, 539·8, 753), and/or an abnormal renal function test with estimated glomerular filtration rate (eGFR) <90 mL/min/1·73 m^2 before the study follow-up or at baseline.

© 2025 Chan JKN et al. *JAMA Network Open*.

Covariates of interest

We selected a comprehensive array of candidate covariates a priori, comprising patient demographics (age at diagnosis, sex), catchment areas of receipt for healthcare service (for geographic and hospital-based variation), pre-existing physical comorbidities as quantified by the Charlson Comorbidity Index (CCI) as well as hypertension, dyslipidemia and diabetes mellitus, substance and alcohol use disorders, and prescriptions of other psychotropics including antipsychotics, other mood-stabilizing anticonvulsants (carbamazepine and lamotrigine), and antidepressants. Use of medications that may interact with lithium with known risk of nephrotoxicity were also included in the analyses for CKD outcomes, comprising nonsteroid anti-inflammatory drugs (NSAIDs) [with exposure >30 days], angiotensin-converting enzyme inhibitors (ACEIs) and angiotensin II receptor blockers (ARBs), and diuretics. Unless otherwise specified, exposure to covariate medications was defined as ≥1 prescription of the specified medication during study follow-up.

Statistical analysis

Incidence rates of hypothyroidism, hyperthyroidism and CKD outcomes

Incidence rates of thyroid and renal outcomes per 1000 person-years were estimated for lithium and non-lithium groups by an exact 95% confidence intervals (CIs) based on a Poisson distribution. Kaplan-Meier curves were plotted to visualize cumulative incidence of the adverse outcomes and compared between lithium and non-lithium groups with log-rank test. The proportional hazards assumption was confirmed according to the scaled Schoenfeld residuals. Similarly, incidence rates of thyroid and renal outcomes per 1000 person-years (with 95% CIs) were evaluated for the four groups of valproate-, quetiapine-, olanzapine- and risperidone-users.

Sensitivity analysis for the main study analyses.

Five sets of sensitivity analyses were performed to examine the robustness of main study results. First, only lithium-users with medication-possession-ratio (MPR) ≥80% were included in the lithium group to ensure adequate treatment adherence and the predominance of exposure to lithium. MPR was calculated by dividing the cumulative lithium-exposure duration by the length of the total follow-up period per individual patient. Second, only lithium-users with lithium as their first-ever mood stabiliser prescribed during the study period were included in the lithium group to limit the confounding effect of prior exposure to other studied mood-stabilising agents. Third, only lithium-users with a cumulative duration of lithium exposure ≥30 days were included in the lithium group to minimize misclassification bias. Fourth, only lithium-users with ≥2 measurements of lithium serum-levels were included in the lithium group. Fifth, only lithium-users with mean lithium serum-level greater than the median of lithium serum-levels of the entire lithium group to ensure the lithium-users included the analysis had been exposed to “higher” lithium doses/levels.

© 2025 Chan JKN et al. *JAMA Network Open*.

References

1. Chan JKN, Wong CSM, Yung NCL, Chen EYH, Chang WC. Excess mortality and life-years lost in people with bipolar disorder: an 11-year population-based cohort study. Epidemiol Psychiatr Sci. 2021;30:e39.
2. Chan JKN, Wong CSM, Or PCF, Chen EYH, Chang WC. Risk of mortality and complications in patients with schizophrenia and diabetes mellitus: population-based cohort study. Br J Psychiatry. 2021;219(1):375-382.
3. Chan JKN, Wong CSM, Or PCF, Chen EYH, Chang WC. Diabetes complication burden and patterns and risk of mortality in people with schizophrenia and diabetes: A population-based cohort study with 16-year follow-up. Eur Neuropsychopharmacol. 2021;53:79-88.
4. Chan JKN, Wong CSM, Yung NCL, Chen EYH, Chang WC. Pre-existing chronic physical morbidity and excess mortality in people with schizophrenia: a population-based cohort study. Soc Psychiatry Psychiatr Epidemiol. 2022;57(3):485-493.
5. Chan JKN, Fang CZ, Lo HKY, Wong CSM, Yung NCL, Chang WC. Pre-existing chronic physical morbidity and excess mortality in people with bipolar disorder: a population-based cohort study in 2008-2018. Eur Neuropsychopharmacol. 2024;84:20.
6. Chang WC, Chan JKN, Wong CSM, Hai JSH, Or PCF, Chen EYH. Mortality, revascularization, and cardioprotective pharmacotherapy after acute coronary syndrome in patients with psychotic disorders: a population-based cohort study. Schizophr Bull. 2020;46(4):774-784.
7. Yung NCL, Wong CSM, Chan JKN, Chen EYH, Chang WC. Excess mortality and life-years lost in people with schizophrenia and other non-affective psychoses: an 11-year population-based cohort study. Schizophr Bull. 2021;47(2):474-484.
8. Chan AYL, Gao L, Howard LM, Simonoff E, Coghill D, Ip P, Lau WCY, Taxis K, Wong ICK, Man KKC. Maternal benzodiazepines and z-drugs use during pregnancy and adverse birth and neurodevelopmental outcomes in offspring: a population-based cohort study. Psychother Psychosom 2023;92(2):113-123.
9. Chan JKN, Wong CSM, Fang CZ, Hung SC, Lo HKY, Chang WC. Mortality risk and mood stabilizers in bipolar disorder: a propensity-score-weighted population-based cohort study in 2002-2018. Epidemiol Psychiatr Sci 2024;33:e31.
10. Chan JKN, Lee KCK, Wong CSM, Chang WC. Risk of congenital malformations associated with first-trimester exposure to antipsychotics: a propensity score-weighted population-based cohort study. Eur Psychiatry 2024;67:e42.
11. Hung C, Chan JKN, Wong CSM, Fung VSC, Lee KCK, Chang WC. Antidepressant utilization patterns and predictors of treatment continuation in pregnant women: A 16-year population-based cohort. Aust N Z J Psychiatry 2023;57(5):686-697.
12. Kan ACO, Chan JKN, Wong CSM, Chen EYH, Chang WC. Psychotropic drug utilization patterns in pregnant women with bipolar disorder: a 16-year population-based cohort study. Eur Neuropsychopharmacol 2022;57:75-85.
13. Law JWY, Chan JKN, Wong CSM, Chen EYH, Chang WC. Antipsychotic utilization patterns in pregnant women with psychotic disorders: a 16-year population-based cohort study. Eur Arch Psychiatry Clin Neurosci 2023;273(4):901-909.

© 2025 Chan JKN et al. *JAMA Network Open*.

14. Lo HKY, Tong CCHY, Chan JKN, Kam CTK, Wong CSM, Cheng CPW, Ho C, Leung BMH, Wong WSH, Yu ZHS, Chang WC. Temporal trends of antidepressant utilization patterns in children and adolescents in Hong Kong: A 14-year population-based study with joinpoint regression analysis. J Affect Disord 2024;344:61-68.
15. Man KKC, Chan EW, Ip P, Coghill D, Simonoff E, Chan PKL, Lau WCY, Schuemie MJ, Sturkenboom MCJM, Wong ICK. Prenatal antidepressant use and risk of attention-deficit/hyperactivity disorder in offspring: population based cohort study. BMJ 2017;357:j2350.
16. Wang Z, Chan AYL, Coghill D, Ip P, Lau WCY, Simonoff E, Brauer R, Wei L, Wong ICK, Man KKC. Association between prenatal exposure to antipsychotics and attention-deficit/hyperactivity disorder, autism spectrum disorder, preterm birth, and small for gestational age. JAMA Intern Med 2021;181(10):1332-1340.
17. Benchimol EI, Smeeth L, Guttmann A, Harron K, Moher D, Petersen I, Sørensen HT, von Elm E, Langan SM; RECORD Working Committee. The REporting of studies Conducted using Observational Routinely-collected health Data (RECORD) Statement. PLoS Med. 2015;12(10):e1001885.

© 2025 Chan JKN et al. *JAMA Network Open*.

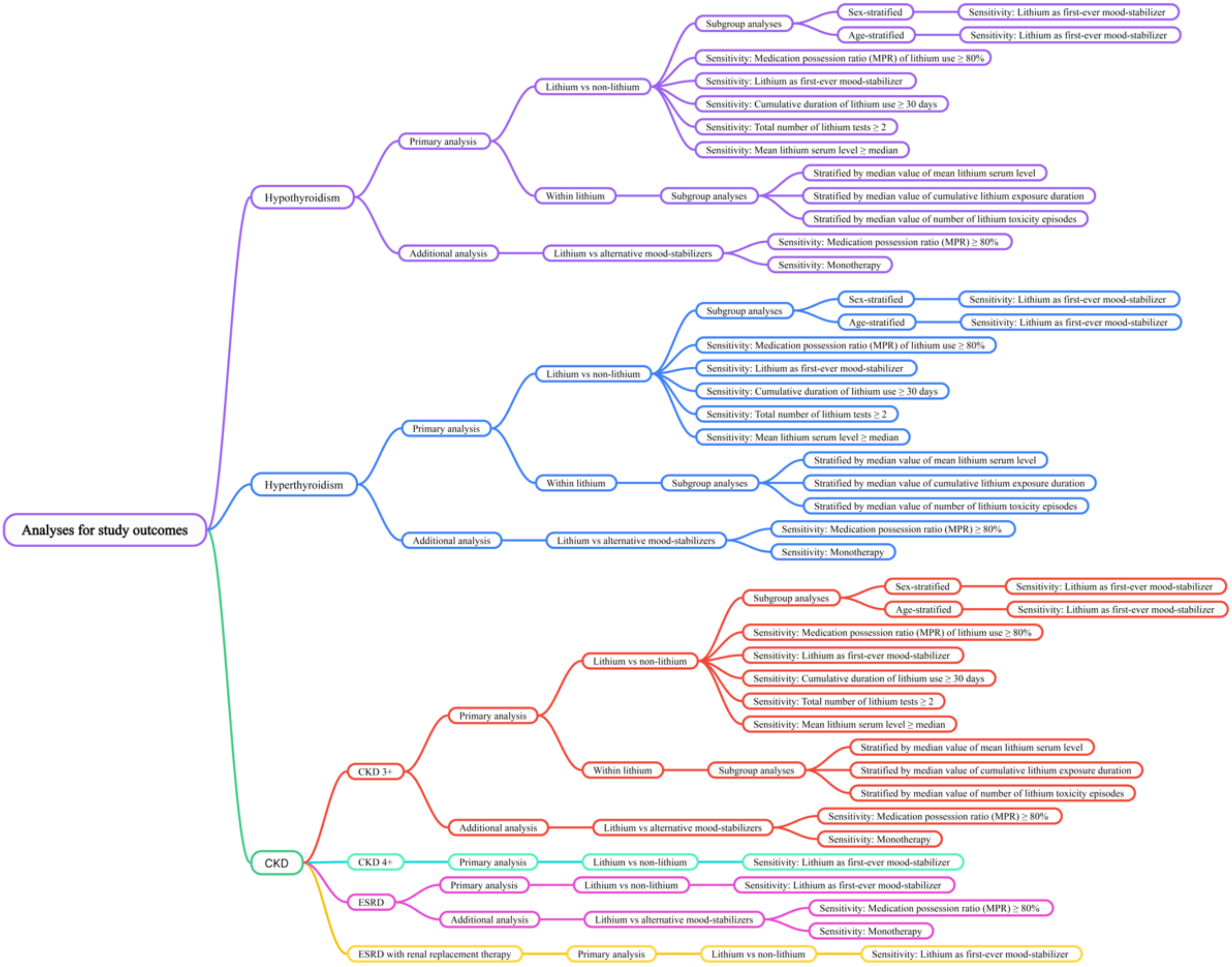

eFigure 1. Flowchart showing primary, additional, subgroup and sensitivity analyses for the risk of hypothyroidism, hyperthyroidism and chronic kidney disease in bipolar disorder patients. CKD, chronic kidney disease; CKD 3+, chronic kidney disease stage 3 or higher; CKD 4+, chronic kidney disease stage 4 or higher; ESRD, end-stage renal disease; MPR, medication possession ratio.

© 2025 Chan JKN et al. *JAMA Network Open*.

eTable 1. Description of study population, exposure, covariates and outcome in subgroup and sensitivity analyses

	Population	Exposure comparison	Covariates
**Hypothyroidism**			
Primary analyses (Lithium versus non-lithium; within lithium group)			
Subgroup analyses			
Sex-stratified subgroup	-Male with bipolar disorder (n=1863)<br>-Female with bipolar disorder (n=2889)	-Lithium (Male: n=746; Female: n=979) versus non-lithium (Male: n=1117; Female: n=1910)	-Age at diagnosis, age-adjusted CCI score, hypertension, dyslipidemia, diabetes mellitus, and prescriptions of other psychotropics including antipsychotics and other mood-stabilizing anticonvulsants (carbamazepine and lamotrigine)
Age-stratified subgroup	-<40 years individuals with bipolar disorder (n=2687)<br>-40–59 years individuals with bipolar disorder (n=1606)<br>-≥60 years individuals with bipolar disorder (n=459)	-Lithium (<40 years: n=1119; 40–59 years: n=545; and ≥60 years: n=61) versus non-lithium (<40 years: n=1568; 40–59 years: n=1061; and ≥60 years: n=398)	-Age at diagnosis, sex, age-adjusted CCI score, hypertension, dyslipidemia, diabetes mellitus, and prescriptions of other psychotropics including antipsychotics and other mood-stabilizing anticonvulsants (carbamazepine and lamotrigine)
Subgroup stratified by median value of mean lithium serum level	-Lithium users (n=1598)	-Within lithium: mean lithium serum level below group median (n=794) versus mean lithium serum level above group median (n=804)	-Age at bipolar disorder diagnosis, sex, age-adjusted CCI score, hypertension, dyslipidemia, diabetes melltius, and prescriptions of other antipsychotics (i.e. antipsychotics other than olanzapine, quetiapine, risperidone), other mood-stabilizing anticonvulsants (i.e. carbamazepine, lamotrigine), any antidepressants, and studied mood-stabilizers other than the specified agent
Subgroup stratified by median value of cumulative lithium exposure duration	-Lithium users (n=1721)	-Within lithium: cumulative lithium exposure duration below group median (n=861) versus cumulative lithium exposure duration above group median (n=860)	-Age at bipolar disorder diagnosis, sex, age-adjusted CCI score, hypertension, dyslipidemia, diabetes mellitus, and prescriptions of other antipsychotics (i.e. antipsychotics other than olanzapine, quetiapine, risperidone), other mood-stabilizing anticonvulsants (i.e. carbamazepine, lamotrigine), any antidepressants,

© 2025 Chan JKN et al. *JAMA Network Open*.

			and studied mood-stabilizers other than the specified agent
Subgroup stratified by median value of number of lithium toxicity episodes	-Lithium users (n=1725)	-Within lithium: number of lithium toxicity episodes below group median (n for toxicity cut-off at 1.0 mmol/L=1365; n for 1.2 mmol/L=1553; n for 1.5 mmol/L=1651) versus number of lithium toxicity episodes above group median (n for toxicity cut-off at 1.0 mmol/L=360; n for 1.2 mmol/L=172; n for 1.5 mmol/L=74)	-Age at bipolar disorder diagnosis, sex, age-adjusted CCI score, hypertension, dyslipidemia, diabetes mellitus, and prescriptions of other antipsychotics (i.e. antipsychotics other than olanzapine, quetiapine, risperidone), other mood-stabilizing anticonvulsants (i.e. carbamazepine, lamotrigine), any antidepressants, and studied mood-stabilizers other than the specified agent
Sensitivity analyses			
Medication possession ratio (MPR) of lithium use ≥ 80%	-Individuals with bipolar disorder (n=4648)	-Lithium MPR ≥ 80% (n=1621) versus non-lithium group (n=3027)	-Lithium versus non-lithium: age at diagnosis, sex, age-adjusted CCI score, hypertension, dyslipidemia, diabetes mellitus, and prescriptions of other psychotropics including antipsychotics and other mood-stabilizing anticonvulsants (carbamazepine and lamotrigine)
Cumulative duration of lithium use ≥ 30 days	-Individuals with bipolar disorder (n=4601)	-Lithium users with cumulative lithium duration ≥ 30 days (n=1574) versus non-lithium group (n=3027)	-Age at diagnosis, sex, age-adjusted CCI score, hypertension, dyslipidemia, diabetes mellitus, and prescriptions of other psychotropics including antipsychotics and other mood-stabilizing anticonvulsants (carbamazepine and lamotrigine)
Total number of lithium tests ≥ 2	-Individuals with bipolar disorder (n=4493)	-Lithium users with total number of lithium tests ≥ 2 (n=1466) versus non-lithium group (n=3027)	-Age at diagnosis, sex, age-adjusted CCI score, hypertension, dyslipidemia, diabetes mellitus, and prescriptions of other psychotropics including antipsychotics and other mood-stabilizing anticonvulsants (carbamazepine and lamotrigine)

© 2025 Chan JKN et al. *JAMA Network Open*.

Mean lithium serum level ≥ median	-Individuals with bipolar disorder (n=3831)	-Lithium users with mean lithium serum level ≥ median (n=804) versus non-lithium group (n=3027)	-Age at diagnosis, sex, age-adjusted CCI score, hypertension, dyslipidemia, diabetes mellitus, and prescriptions of other psychotropics including antipsychotics and other mood-stabilizing anticonvulsants (carbamazepine and lamotrigine)
Lithium as first-ever mood-stabilizer	-Individuals with bipolar disorder (n=3416)	-Lithium users with lithium as the first-ever mood-stabilizer (n=389) versus non-lithium group (n=3027)	-Age at diagnosis, sex, age-adjusted CCI score, hypertension, dyslipidemia, diabetes mellitus, and prescriptions of other psychotropics including antipsychotics and other mood-stabilizing anticonvulsants (carbamazepine and lamotrigine)
Additional analyses (Lithium versus alternative mood-stabilizers)			
Sensitivity analyses			
MPR of ≥ 80%	-Individuals with bipolar disorder (n=4648)	-Lithium (reference; n=1621) versus valproate (n=1521), olanzapine (n=268), quetiapine (n=627) and risperidone (n=261)	-Age at bipolar disorder diagnosis, sex, catchment area of psychiatric service receipt, hypertension, dyslipidemia, diabetes mellitus, alcohol and substance use disorders, age-adjusted CCI score as well as prescription records of other antipsychotics (i.e. antipsychotics other than olanzapine, quetiapine, risperidone), other mood-stabilizing anticonvulsants (i.e. carbamazepine, lamotrigine), any antidepressants, and studied mood-stabilizers other than the specified agent
Monotherapy	-Individuals with bipolar disorder (n=856)	-Lithium (reference; n=146) versus valproate (n=397), olanzapine (n=37), quetiapine (n=199) and risperidone (n=77)	-Age at bipolar disorder diagnosis, sex, catchment area of psychiatric service receipt, age-adjusted CCI score, hypertension, dyslipidemia, diabetes mellitus, alcohol and substance use disorders, prescription records of other antipsychotics (i.e. antipsychotics other than olanzapine, quetiapine, risperidone), other mood-

© 2025 Chan JKN et al. *JAMA Network Open*.

			stabilizing anticonvulsants (i.e. carbamazepine, lamotrigine), any antidepressants, and studied mood-stabilizers other than the specified agent
**Hyperthyroidism**			
Primary analyses (Lithium vs non-lithium; within lithium group)			
Subgroup analyses			
Sex-stratified subgroup	-Male with bipolar disorder (n=1784)<br>-Female with bipolar disorder (n=2716)	-Lithium (Male: n=687; Female: n=851) versus non-lithium (Male: n=1097; Female: n=1865)	-Age at diagnosis, age-adjusted CCI score, hypertension, dyslipidemia, diabetes mellitus, and prescriptions of other psychotropics including antipsychotics and other mood-stabilizing anticonvulsants (carbamazepine and lamotrigine)
Age-stratified subgroup	-<40 years individuals with bipolar disorder (n=2530)<br>-40–59 years individuals with bipolar disorder (n=1525)<br>-≥60 years individuals with bipolar disorder (n=445)	-Lithium (<40 years: n=999; 40–59 years: n=483; ≥60 years: n=56) versus non-lithium (<40 years: n=1531; 40–59 years: n=1042; ≥60 years: n=389)	-Age at diagnosis, sex, age-adjusted CCI score, hypertension, dyslipidemia, diabetes mellitus, and prescriptions of other psychotropics including antipsychotics and other mood-stabilizing anticonvulsants (carbamazepine and lamotrigine)
Subgroup stratified by median value of mean lithium serum level	-Lithium users (n=1413)	-Within lithium: mean lithium serum level below group median (n=701) versus mean lithium serum level above group median (n=712)	-Age at bipolar disorder diagnosis, sex, age-adjusted CCI score, hypertension, dyslipidemia, diabetes mellitus, and prescriptions of other antipsychotics (i.e. antipsychotics other than olanzapine, quetiapine, risperidone), other mood-stabilizing anticonvulsants (i.e. carbamazepine, lamotrigine), any antidepressants, and studied mood-stabilizers other than the specified agent
Subgroup stratified by median value of cumulative lithium exposure duration	-Lithium users (n=1535)	-Within lithium: cumulative lithium exposure duration below group median (n=768) versus cumulative lithium exposure duration above group median (n=767)	-Age at bipolar disorder diagnosis, sex, age-adjusted CCI score, hypertension, dyslipidemia, diabetes mellitus, and prescriptions of other antipsychotics (i.e. antipsychotics

© 2025 Chan JKN et al. *JAMA Network Open*.

			other than olanzapine, quetiapine, risperidone), other mood-stabilizing anticonvulsants (i.e. carbamazepine, lamotrigine), any antidepressants, and studied mood-stabilizers other than the specified agent
Subgroup stratified by median value of number of lithium toxicity episodes	-Lithium users (n=1538)	-Within lithium: number of lithium toxicity episodes below group median (n for toxicity cut-off at 1.0 mmol/L=1212; n for 1.2 mmol/L=1381; n for 1.5 mmol/L=1465) versus number of lithium toxicity episodes above group median (n for toxicity cut-off at 1.0 mmol/L=326; n for 1.2 mmol/L=157; n for 1.5 mmol/L=73)	-Age at bipolar disorder diagnosis, sex, age-adjusted CCI score, hypertension, dyslipidemia, diabetes mellitus, and prescriptions of other antipsychotics (i.e. antipsychotics other than olanzapine, quetiapine, risperidone), other mood-stabilizing anticonvulsants (i.e. carbamazepine, lamotrigine), any antidepressants, and studied mood-stabilizers other than the specified agent
Sensitivity analyses			
MPR of lithium use ≥ 80%	-Individuals with bipolar disorder (n=4448)	-Lithium MPR ≥ 80% (n=1486) versus non-lithium group (n=2962)	-Age at diagnosis, sex, age-adjusted CCI score, hypertension, dyslipidemia, diabetes mellitus, and prescriptions of other psychotropics including antipsychotics and other mood-stabilizing anticonvulsants (carbamazepine and lamotrigine)
Cumulative duration of lithium use ≥ 30 days	-Individuals with bipolar disorder (n=4389)	-Lithium users with cumulative lithium duration ≥ 30 days (n=1427) versus non-lithium group (n=2962)	-Age at diagnosis, sex, age-adjusted CCI score, hypertension, dyslipidemia, diabetes mellitus, and prescriptions of other psychotropics including antipsychotics and other mood-stabilizing anticonvulsants (carbamazepine and lamotrigine)
Total number of lithium tests ≥ 2	-Individuals with bipolar disorder (n=4428)	-Lithium users with total number of lithium tests ≥ 2 (n=1466) versus non-lithium group (n=2962)	-Age at diagnosis, sex, age-adjusted CCI score, hypertension, dyslipidemia, diabetes mellitus, and prescriptions of other psychotropics including antipsychotics and other

© 2025 Chan JKN et al. *JAMA Network Open*.

			mood-stabilizing anticonvulsants (carbamazepine and lamotrigine)
Mean lithium serum level ≥ median	-Individuals with bipolar disorder (n=3766)	-Lithium users with mean lithium serum level ≥ median (n=804) versus non-lithium group (n=2962)	-Age at diagnosis, sex, age-adjusted CCI score, hypertension, dyslipidemia, diabetes mellitus, and prescriptions of other psychotropics including antipsychotics and other mood-stabilizing anticonvulsants (carbamazepine and lamotrigine)
Lithium as first-ever mood-stabilizer	-Individuals with bipolar disorder (n=3339)	-Lithium users with lithium as the first-ever mood-stabilizer (n=377) versus non-lithium group (n=2962)	-Age at diagnosis, sex, age-adjusted CCI score, hypertension, dyslipidemia, diabetes mellitus, and prescriptions of other psychotropics including antipsychotics and other mood-stabilizing anticonvulsants (carbamazepine and lamotrigine)
Additional analyses (Lithium versus alternative mood-stabilizers)			
Sensitivity analyses			
MPR ≥ 80%	-Individuals with bipolar disorder (n=4448)	-Lithium (reference; n=1486) versus valproate (n=1459), olanzapine (n=259), quetiapine (n=614) and risperidone (n=270)	-Age at bipolar disorder diagnosis, sex, catchment area of psychiatric service receipt, hypertension, dyslipidemia, diabetes mellitus, alcohol and substance use disorders, age-adjusted CCI score as well as prescription records of other antipsychotics (i.e. antipsychotics other than olanzapine, quetiapine, risperidone), other mood-stabilizing anticonvulsants (i.e. carbamazepine, lamotrigine), any antidepressants, and studied mood-stabilizers other than the specified agent
Monotherapy	-Individuals with bipolar disorder (n=831)	-Lithium (reference; n=124) versus valproate (n=381), olanzapine (n=36), quetiapine (n=205) and risperidone (n=85)	-Age at bipolar disorder diagnosis, sex, catchment area of psychiatric service receipt, age-adjusted CCI score, hypertension, dyslipidemia, diabetes mellitus, alcohol and substance use disorders, prescription

© 2025 Chan JKN et al. *JAMA Network Open*.

			records of other antipsychotics (i.e. antipsychotics other than olanzapine, quetiapine, risperidone), other mood-stabilizing anticonvulsants (i.e. carbamazepine, lamotrigine), any antidepressants, and studied mood-stabilizers other than the specified agent
**CKD stage 3 or higher**			
Primary analyses (Lithium vs non-lithium; within lithium group)			
Subgroup analyses			
Sex-stratified subgroup	-Male with bipolar disorder (n=2778)<br>-Female with bipolar disorder (n=4251)	-Lithium (Male: n=930; Female: n=1328) versus non-lithium (Male: n=1848; Female: n=2923)	-Age at bipolar disorder diagnosis, catchment area of psychiatric service receipt, age-adjusted CCI score, hypertension, dyslipidemia, diabetes mellitus, and prescriptions of other antipsychotics, mood-stabilizing anticonvulsants, antidepressant, NSAIDs, ACEIs/ARBs and diuretics
Age-stratified subgroup	-<40 years individuals with bipolar disorder (n=2687)<br>-40–59 years individuals with bipolar disorder (n=1606)<br>-≥60 years individuals with bipolar disorder (n=459)	-Lithium (<40 years: n=1119; 40–59 years: n=545; ≥60 years: n=61) versus non-lithium (<40 years: n=1568; 40–59 years: n=1061; ≥60 years: n=398)	-Age at bipolar disorder diagnosis, catchment area of psychiatric service receipt, age-adjusted CCI score, hypertension, dyslipidemia, diabetes mellitus, and prescriptions of other antipsychotics, mood-stabilizing anticonvulsants, antidepressant, NSAIDs, ACEIs/ARBs and diuretics
Subgroup stratified by median value of mean lithium serum level	-Lithium users (n=2084)	-Within lithium: mean lithium serum level below group median (n=1049) versus mean lithium serum level above group median (n=1035)	-Age at bipolar disorder diagnosis, sex, age-adjusted CCI score, hypertension, dyslipidemia, diabetes mellitus, catchment area of psychiatric service receipt, and prescriptions of other antipsychotics, mood-stabilizing anticonvulsants, antidepressant, NSAIDs, ACEIs/ARBs and diuretics

© 2025 Chan JKN et al. *JAMA Network Open*.

Subgroup stratified by median value of cumulative lithium exposure duration	-Lithium users (n=2030)	-Within lithium: mean lithium serum level below group median (n=903) versus mean lithium serum level above group median (n=1127)	-Age at bipolar disorder diagnosis, sex, age-adjusted CCI score, hypertension, dyslipidemia, diabetes mellitus, catchment area of psychiatric service receipt, and prescriptions of other antipsychotics, mood-stabilizing anticonvulsants, antidepressant, NSAIDs, ACEIs/ARBs and diuretics
Subgroup stratified by median value of number of lithium toxicity episodes	-Lithium users (n=2258)	-Within lithium: number of lithium toxicity episodes below group median (n for toxicity cut-off at 1.0 mmol/L=1716; n for 1.2 mmol/L=2013; n for 1.5 mmol/L=2161) versus number of lithium toxicity episodes above group median (n for toxicity cut-off at 1.0 mmol/L=542; n for 1.2 mmol/L=245; n for 1.5 mmol/L=97)	-Age at bipolar disorder diagnosis, sex, age-adjusted CCI score, hypertension, dyslipidemia and diabetes mellitus, catchment area of psychiatric service receipt, and prescriptions of other antipsychotics, mood-stabilizing anticonvulsants, antidepressant, NSAIDs, ACEIs/ARBs and diuretics
Sensitivity analyses			
MPR of lithium use ≥ 80%	-Individuals with bipolar disorder (n=6865)	-Lithium MPR ≥ 80% (n=2094) versus non-lithium group (n=4771)	-Age at bipolar disorder diagnosis, catchment area of psychiatric service receipt, age-adjusted CCI score, hypertension, dyslipidemia, diabetes mellitus, and prescriptions of other antipsychotics, mood-stabilizing anticonvulsants, antidepressant, NSAIDs, ACEIs/ARBs and diuretics
Cumulative duration of lithium use ≥ 30 days	-Individuals with bipolar disorder (n=4389)	-Lithium users with cumulative lithium duration ≥ 30 days (n=2081) versus non-lithium group (n=2962)	-Age at diagnosis, sex, age-adjusted CCI score, hypertension, dyslipidemia, diabetes mellitus, and prescriptions of other psychotropics including antipsychotics and other mood-stabilizing anticonvulsants (carbamazepine and lamotrigine)
Total number of lithium tests ≥ 2	-Individuals with bipolar disorder (n=6674)	-Lithium users with total number of lithium tests ≥ 2 (n=1903) versus non-lithium group (n=4771)	-Age at diagnosis, sex, age-adjusted CCI score, hypertension, dyslipidemia, diabetes mellitus, and prescriptions of other psychotropics

© 2025 Chan JKN et al. *JAMA Network Open*.

			including antipsychotics and other mood-stabilizing anticonvulsants (carbamazepine and lamotrigine)
Mean lithium serum level ≥ median	-Individuals with bipolar disorder (n=5806)	-Lithium users with mean lithium serum level ≥ median (n=1035) versus non-lithium group (n=4771)	-Age at diagnosis, sex, age-adjusted CCI score, hypertension, dyslipidemia, diabetes mellitus, and prescriptions of other psychotropics including antipsychotics and other mood-stabilizing anticonvulsants (carbamazepine and lamotrigine)
Lithium as first-ever mood-stabilizer	-Individuals with bipolar disorder (n=5326)	-Lithium users with lithium as the first-ever mood-stabilizer (n=555) versus non-lithium group (n=4771)	-Age at diagnosis, sex, age-adjusted CCI score, hypertension, dyslipidemia, diabetes mellitus, and prescriptions of other psychotropics including antipsychotics and other mood-stabilizing anticonvulsants (carbamazepine and lamotrigine)
Additional analyses (Lithium versus alternative mood-stabilizers)			
Sensitivity analyses			
MPR ≥ 80%	-Individuals with bipolar disorder (n=6865)	-Lithium (reference; n=2094) versus valproate (n=2399), olanzapine (n=364), quetiapine (n=966) and risperidone (n=388)	-Age at bipolar disorder diagnosis, sex, catchment area of psychiatric service receipt, age-adjusted CCI score, hypertension, dyslipidemia, diabetes mellitus, alcohol and substance use disorders, and prescription of other antipsychotics, other mood-stabilizing anticonvulsants, any antidepressants, and studied mood-stabilizers other than the specified agent, NSAIDs, ACEIs/ARBs and diuretics
Monotherapy	-Individuals with bipolar disorder (n=1466)	-Lithium (reference; n=184) versus valproate (n=694), olanzapine (n=74), quetiapine (n=375) and risperidone (n=139)	-Age at BD diagnosis, sex, catchment area of service receipt, age-adjusted CCI score, hypertension, dyslipidemia, diabetes mellitus, alcohol and substance abuse, and prescription of other antipsychotics, other mood-stabilizing anticonvulsants, any

© 2025 Chan JKN et al. *JAMA Network Open*.

			antidepressants and studied mood-stabilizers other than the specified agent, as well as exposure to NSAIDs, ACEI/ARB, diuretics
**CKD stage 4 or higher**			
Primary analyses (Lithium vs non-lithium)			
Sensitivity analysis			
Lithium as first-ever mood-stabilizer	-Individuals with bipolar disorder (n=5326)	-Lithium users with lithium as the first-ever mood-stabilizer (n=555) versus non-lithium group (n=4771)	-Age at diagnosis, sex, age-adjusted CCI score, hypertension, dyslipidemia, diabetes mellitus, and prescriptions of other psychotropics including antipsychotics and other mood-stabilizing anticonvulsants (carbamazepine and lamotrigine)
**ESRD**			
Primary analyses (Lithium vs non-lithium)			
Sensitivity analyses			
Lithium as first-ever mood-stabilizer	-Individuals with bipolar disorder (n=5326)	-Lithium users with lithium as the first-ever mood-stabilizer (n=555) versus non-lithium group (n=4771)	-Age at diagnosis, sex, age-adjusted CCI score, hypertension, dyslipidemia, diabetes mellitus, and prescriptions of other psychotropics including antipsychotics and other mood-stabilizing anticonvulsants (carbamazepine and lamotrigine)
Additional analyses (Lithium vs alternative mood-stabilizers)			
Sensitivity analyses			
MPR ≥ 80%	-Individuals with bipolar disorder (n=6309)	-Lithium (reference; n=2175) versus valproate (n=2407), olanzapine (n=365), quetiapine (n=972) and risperidone (n=390)	-Age at bipolar disorder diagnosis, sex, catchment area of psychiatric service receipt, age-adjusted CCI score, hypertension, dyslipidemia, diabetes mellitus, alcohol and substance use disorders, and prescription of other antipsychotics, other mood-stabilizing anticonvulsants, any antidepressants, and studied mood-stabilizers other than the specified agent, NSAIDs, ACEIs/ARBs and diuretics
**ESRD with renal replacement therapy**			

© 2025 Chan JKN et al. *JAMA Network Open*.

Primary analysis (Lithium vs non-lithium)			
Sensitivity analysis			
Lithium as first-ever mood-stabilizer	-Individuals with bipolar disorder (n=5326)	-Lithium users with lithium as the first-ever mood-stabilizer (n=555) versus non-lithium group (n=4771)	-Age at diagnosis, sex, age-adjusted CCI score, hypertension, dyslipidemia, diabetes mellitus, and prescriptions of other psychotropics including antipsychotics and other mood-stabilizing anticonvulsants (carbamazepine and lamotrigine)
Abbreviations: ACEIs, angiotensin-converting-enzyme inhibitors; ARBs, angiotensin receptor blockers; BD, bipolar disorder; CCI, Charlson comorbidity index; CKD, chronic kidney disease; ESRD, end-stage renal disease; MPR, medication possession ratio; NSAIDs, non-steroidal anti-inflammatory drugs.			

© 2025 Chan JKN et al. *JAMA Network Open*.

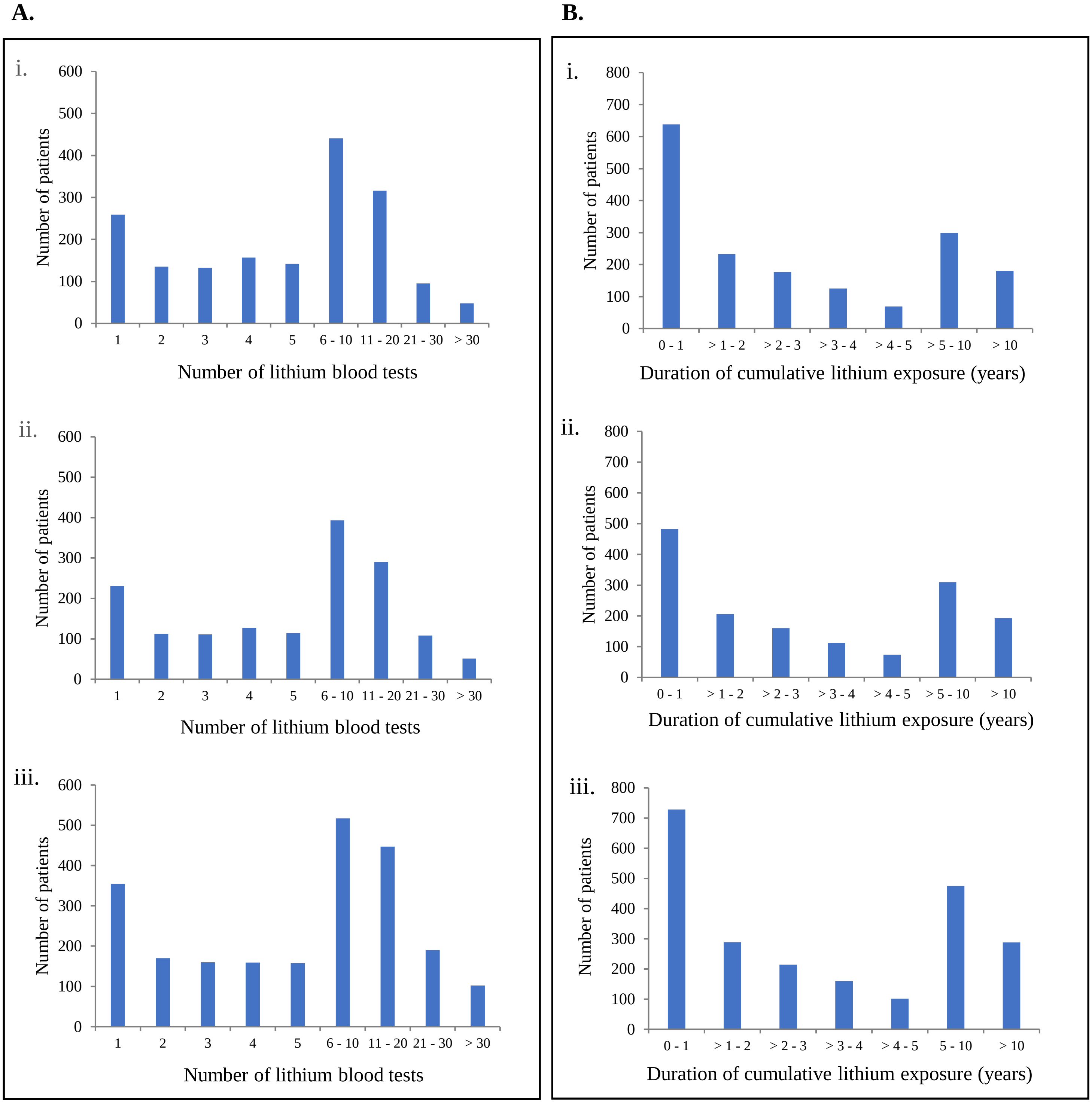

eFigure 2. (A) Number of lithium blood tests of lithium users, and (B) duration of cumulative exposure in lithium users in the study samples for (i) hypothyroidism, (ii) hyperthyroidism, and (iii) chronic kidney disease.

© 2025 Chan JKN et al. *JAMA Network Open*.

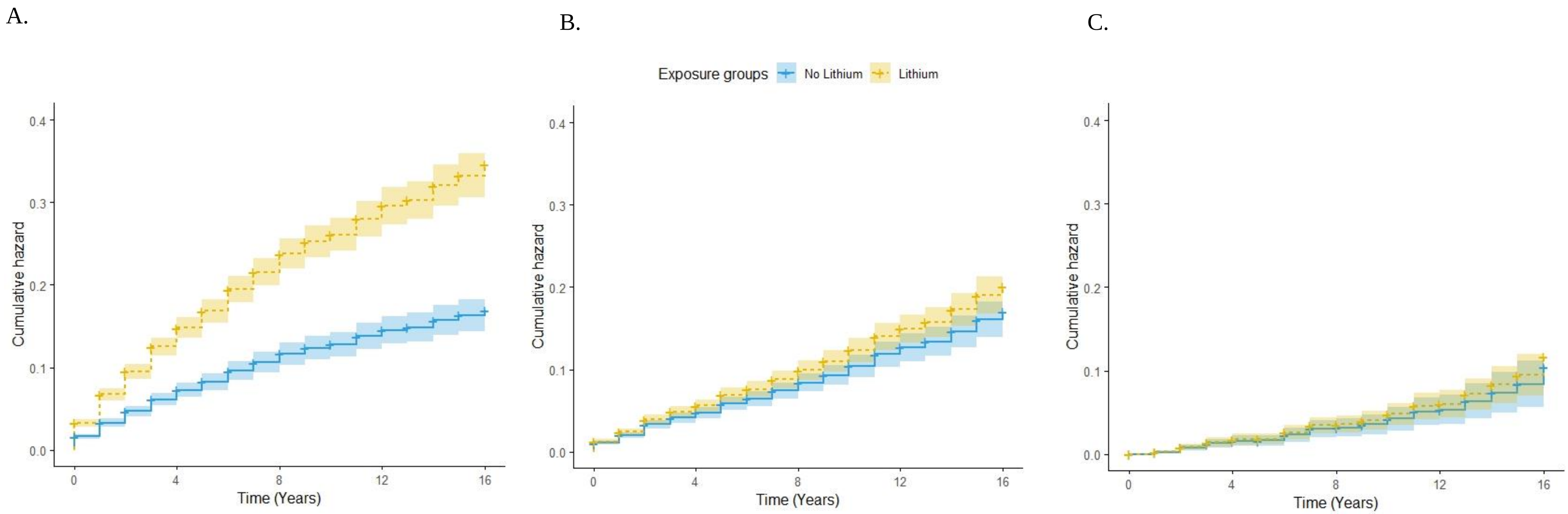

eFigure 3. Cumulative hazard for (A) hypothyroidism, (B) hyperthyroidism and (C) chronic kidney disease in BD patients with versus without lithium use. Chronic kidney disease is defined as stage 3 or above. BD, bipolar disorder.

© 2025 Chan JKN et al. *JAMA Network Open*.

eTable 2. Sensitivity analyses on association between lithium use and risk of hypothyroidism, hyperthyroidism and chronic kidney disease

Sensitivity analyses	BD patients with lithium use<br>Event/ Total sample	Adjusted HR (95% CI)	P [a]
Hypothyroidism			
Medication possession ratio ≥ 80%	306/ 1621	1.63 (1.27 – 2.10)	<0.001 [b]
Cumulative duration ≥ 30 days	312/ 1574	1.83 (1.56 – 2.15)	<0.001 [b]
Total number of lithium tests ≥ 2	312/ 1466	1.97 (1.68 – 2.31)	<0.001 [b]
Mean lithium serum level ≥ median	220/ 804	2.58 (2.17 – 3.08)	<0.001 [b]
Hyperthyroidism			
Medication possession ratio ≥ 80%	171/ 1486	1.27 (0.96 – 1.68)	0.09
Cumulative duration ≥ 30 days	165/ 1427	1.15 (0.94 – 1.40)	0.19
Total number of lithium tests ≥ 2	153/ 1307	1.14 (0.93 – 1.40)	0.21
Mean lithium serum level ≥ median	103/ 712	1.38 (1.09 – 1.74)	0.007
CKD 3+			
Medication possession ratio ≥ 80%	197/ 2094	1.78 (1.41 – 2.26)	<0.001 [b]
Cumulative duration ≥ 30 days	206/ 2081	1.33 (1.12 – 1.58)	0.001 [b]
Total number of lithium tests ≥ 2	188/ 1903	1.42 (1.19 – 1.70)	<0.001 [b]
Mean lithium serum level ≥ median	139/ 1035	1.82 (1.50 – 2.22)	<0.001 [b]

Abbreviations: BD, bipolar disorder; CKD, chronic kidney disease; CI, confidence intervals; HR, hazard ratio. [a] Bonferroni correction for multiple comparison was applied (corrected p-value=0.00417). [b] Results remained significant after Bonferroni correction.

© 2025 Chan JKN et al. *JAMA Network Open*.

eTable 3. Association between lithium use as first-ever mood-stabilizer and risk of hypothyroidism, hyperthyroidism and chronic kidney disease

	BD patients with lithium use			BD patients without lithium use				
	n	Event/ PY	Incidence per 1,000 PY (95% CI)	n	Event/ PY	Incidence per 1,000 PY (95% CI)	Adjusted HR (95% CI)	P [a]
Hypothyroidism	389	72/ 3227	22.3 (17.8 – 28.0)	3027	340/ 25486	13.3 (12.0 – 14.8)	1.49 (1.11 – 2.00)	0.008
Male	164	26/ 1404	18.5 (12.7 – 27.1)	1117	106/ 9199	11.5 (9.5 – 13.9)	1.36 (0.82 – 2.25)	0.23
< 40 years	84	14/ 728	19.2 (11.4 – 32.3)	553	48/ 4734	10.1 (7.7 – 13.4)	1.79 (0.91 – 3.50)	0.09
40 – 59 years	73	12/ 637	18.8 (10.8 – 33.0)	381	35/ 3298	10.6 (7.6 – 14.8)	1.05 (0.46 – 2.45)	0.90
≥ 60 years	7	0/ 39	NA	183	23/ 1167	19.7 (13.2 – 29.5)	NA	0.98
Female	225	46/ 1823	25.2 (19.0 – 33.6)	1910	234/ 16287	14.4 (12.7 – 16.3)	1.55 (1.08 – 2.24)	0.02
< 40 years	127	31/ 1068	29.0 (20.5 – 41.1)	1015	130 /8820	14.7 (12.4 – 17.5)	1.63 (1.03 – 2.58)	0.04
40 – 59 years	83	12/ 662	18.1 (10.3 – 31.8)	680	81 /5817	13.9 (11.2 – 17.3)	1.17 (0.55 – 2.52)	0.68
≥ 60 years	15	3/ 93	32.3 (10.6 – 98.2)	215	23/ 1650	13.9 (9.3 – 20.9)	4.38 (1.29 – 14.85)	0.02
Hyperthyroidism	377	60/ 3337	18.0 (14.0 – 23.1)	2962	275/ 25125	10.9 (9.7 – 12.3)	1.38 (1.03 – 1.85)	0.03
Male	166	28/ 1494	18.7 (13.0 – 27.0)	1097	86/ 9140	9.4 (7.6 – 11.6)	1.57 (0.99 – 2.48)	0.054
< 40 years	83	13/ 773	16.8 (9.8 – 28.8)	547	42/ 4740	8.9 (6.6 – 12.0)	1.49 (0.75 – 2.95)	0.26
40 – 59 years	75	14/ 680	20.6 (12.3 – 34.6)	375	29/ 3217	9.0 (6.3 – 13.0)	1.91 (0.93 – 3.93)	0.08
≥ 60 years	8	1/ 41	71.4 (10.8 – 472.2)	175	15/ 1183	12.7 (7.7 – 21.0)	0.92 (0.09 – 9.22)	0.95
Female	211	32/ 1843	17.4 (12.3 – 24.5)	1865	189/15985	11.8 (10.3 – 13.6)	1.24 (0.84 – 1.83)	0.28
< 40 years	115	19/ 1104	17.2 (11.0 – 26.9)	984	99/ 8667	11.4 (9.4 – 13.9)	1.29 (0.75 – 2.21)	0.37
40 – 59 years	80	9/ 649	13.9 (7.2 – 26.5)	667	68/ 5680	12.0 (9.5 – 15.2)	0.58 (0.25 – 1.38)	0.22
≥ 60 years	16	4/ 90	44.4 (17.1 – 115.8)	214	22/ 1638	13.4 (8.9 – 20.3)	4.83 (1.18 – 19.8)	0.03
CKD 3+ [a]	555	83/ 4844	17.1 (13.8 – 21.2)	4771	527/ 37609	14.0 (12.9 – 15.3)	1.63 (1.28 – 2.07)	<0.001 [b]
Male	229	37/ 1998	18.5 (13.5 – 25.5)	1848	238/ 13995	17.0 (15.0 – 19.3)	1.40 (0.97 – 2.01)	0.07
< 40 years	118	14/ 1113	12.6 (7.5 – 21.2)	922	39/ 7686	5.1 (3.7 – 6.9)	2.18 (1.14 – 4.19)	0.02
40 – 59 years	104	22/ 852	25.8 (17.1 – 39.0)	698	96/ 5219	18.4 (15.01 – 22.4)	1.24 (0.77 – 2.00)	0.38
≥ 60 years	7	1/ 33	30.3 (4.4 – 208.8)	228	103/ 1090	94.5 (78.6 – 113.6)	0.36 (0.05 – 2.74)	0.32
Female	326	46/ 2846	16.2 (12.1 – 21.5)	2923	289/ 23614	12.2 (10.9 – 13.7)	1.83 (1.32 – 2.52)	<0.001 [b]
< 40 years	182	18/ 1774	10.1 (6.4 – 16.1)	1527	48/ 13238	3.6 (2.7 – 4.8)	2.91 (1.66 – 5.11)	<0.001 [b]
40 – 59 years	122	19/ 974	19.5 (12.5 – 30.4)	1129	134/ 8920	15.0 (12.7 – 17.8)	1.35 (0.82 – 2.23)	0.24
≥ 60 years	22	9/ 98	91.8 (49.3 – 171.2)	267	107/ 1456	73.5 (61.2 – 88.2)	2.12 (0.99 – 4.54)	0.052
CKD 4+	555	14/ 5171	2.7 (1.6 – 4.6)	4771	114/ 39757	2.9 (2.4 – 3.4)	1.37 (0.78 – 2.42)	0.27
ESRD	555	4/ 5226	0.8 (0.3 – 2.0)	4771	33/ 39962	0.8 (0.6 – 1.2)	1.31 (0.45 – 3.79)	0.62
ESRD with renal replacement therapy	555	2/ 5232	0.4 (0.1 – 1.5)	4771	16/ 40008	0.4 (0.2 – 0.7)	1.13 (0.25 – 5.13)	0.87

Abbreviations: BD, bipolar disorder; CKD, chronic kidney disease; CI, confidence intervals; ESRD, end-stage renal diseases; PY, person years. CKD was defined as occurrence of ≥CKD stage 3. [a] Bonferroni correction for multiple comparison was applied (corrected p-value=0.00167). [b] Results remained significant after Bonferroni correction.

© 2025 Chan JKN et al. *JAMA Network Open*.

eTable 4. Sensitivity analyses on risk of hypothyroidism, hyperthyroidism and chronic kidney diseases in bipolar disorder people with lithium and other mood-stabilizers

Mood-stabilizing medications	Events/ Total n	Adjusted HR (95% CI)	P [c]
Medication possession ratio ≥80%			
Hypothyroidism [a] (n=4298)			
Lithium	306/ 1621	1 (reference)	-
Valproate	161/ 1521	0.50 (0.41 – 0.61)	<0.001 [d]
Olanzapine	15/ 268	0.29 (0.17 – 0.49)	<0.001 [d]
Quetiapine	55/ 627	0.50 (0.37 – 0.67)	<0.001 [d]
Risperidone	20/ 261	0.37 (0.23 – 0.59)	<0.001 [d]
Hyperthyroidism [a] (n=4088)			
Lithium	171/ 1486	1 (reference)	-
Valproate	99/ 1459	0.54 (0.42 – 0.70)	<0.001 [d]
Olanzapine	6/ 259	0.24 (0.11 – 0.54)	0.001 [d]
Quetiapine	42/ 614	0.70 (0.49 – 1.01)	0.053
Risperidone	29/ 270	0.93 (0.62 – 1.39)	0.71
CKD 3+ [b] (n=6211)			
Lithium	197/ 2094	1 (reference)	-
Valproate	225/ 2399	0.78 (0.63 – 0.97)	0.03
Olanzapine	20/ 364	0.70 (0.44 – 1.11)	0.13
Quetiapine	69/ 966	0.68 (0.50 – 0.91)	0.01
Risperidone	35/ 388	0.75 (0.52 – 1.09)	0.14
ESRD [b] (n=6309)			
Lithium	12/ 2175	1 (reference)	-
Valproate	11/ 2407	0.75 (0.13 – 4.46)	0.76
Olanzapine	2/ 365	2.33 (0.25 – 21.96)	0.46
Quetiapine	7/ 972	1.56 (0.25 – 9.54)	0.63
Risperidone	2/ 390	0.70 (0.08 – 5.90)	0.74
Monotherapy			
Hypothyroidism [a] (n=856)			
Lithium	45/ 146	1 (reference)	-
Valproate	57/ 397	0.45 (0.30 – 0.67)	<0.001 [d]
Olanzapine	2/ 37	0.15 (0.04 – 0.62)	0.009
Quetiapine	17/ 199	0.33 (0.18 – 0.58)	<0.001 [d]
Risperidone	12/ 77	0.49 (0.26 – 0.93)	0.03
Hyperthyroidism [a] (n=831)			
Lithium	23/ 124	1 (reference)	-
Valproate	41/ 381	0.56 (0.33 – 0.95)	0.03
Olanzapine	1/ 36	0.16 (0.02 – 1.17)	0.07
Quetiapine	23/ 205	0.68 (0.37 – 1.25)	0.21
Risperidone	20/ 85	1.20 (0.64 – 2.25)	0.58
CKD 3+ [b] (n=1466)			
Lithium	45/ 184	1 (reference)	-

© 2025 Chan JKN et al. *JAMA Network Open*.

Valproate	118/ 694	0.53 (0.37 – 0.76)	0.001 [d]
Olanzapine	6/ 74	0.40 (0.17 – 0.96)	0.04
Quetiapine	35/ 375	0.39 (0.24 – 0.62)	<0.001 [d]
Risperidone	18/ 139	0.54 (0.31 – 0.95)	0.03
ESRD [b] (n=1466)			
Lithium	3/ 184	1 (reference)	-
Valproate	8/ 694	0.90 (0.17 – 4.63)	0.90
Olanzapine	0/ 74	NA	NA
Quetiapine	2/ 375	0.57 (0.08 – 4.16)	0.58
Risperidone	1/ 139	1.15 (0.09 – 14.19)	0.91

Abbreviations: ACEIs, angiotensin-converting enzyme inhibitors; ARBs, angiotensin receptor blockers; BD, bipolar disorder; CKD, chronic kidney disease; CI, confidence intervals; ESRD, end-stage renal diseases; HR, hazard ratio. NA, not applicable; NASIDs, nonsteroidal anti-inflammatory drugs; PY, person years. [a] Regression models were adjusted for age at bipolar disorder diagnosis, sex, catchment area of psychiatric service receipt, hypertension, dyslipidemia, diabetes, alcohol and substance abuse, age-adjusted CCI score as well as prescription records of other antipsychotics (i.e., antipsychotics other than olanzapine, quetiapine and risperidone), other mood-stabilizing anticonvulsants (i.e., carbamazepine and lamotrigine), any antidepressants, and studied mood-stabilizers other than the specified agent. [b] Regression models were adjusted for age at BD diagnosis, sex, catchment area of service receipt, hypertension, dyslipidemia, diabetes, alcohol and substance abuse, age-adjusted CCI score, and prescription of other antipsychotics, other mood-stabilizing anticonvulsants, any antidepressants and studied mood-stabilizers other than the specified agent, as well as exposure to NSAIDs, ACEI/ARB, diuretics. [c] Bonferroni correction for multiple comparison was applied (corrected p-value=0.0031). [d] Results remained significant after Bonferroni correction.

© 2025 Chan JKN et al. *JAMA Network Open*.
