# Supplementary material for: Lithium for Bipolar Disorder and Risk of Thyroid Dysfunction and Chronic Kidney Disease
Source: JAMA Netw Open. 2025 Feb 11;8(2):e2458608. doi: 10.1001/jamanetworkopen.2024.58608 (PMC11815528; doi:10.1001/jamanetworkopen.2024.58608)
Supplement: Supplement 2. — Data Sharing Statement [file jamanetwopen-e2458608-s002.pdf]

Data Sharing Statement

Chan. Lithium for Bipolar Disorder and Risk of Thyroid Dysfunction and Chronic Kidney Disease. *JAMA Netw Open*. Published February 07, 2025.
doi:10.1001/jamanetworkopen.2024.58608

Data
Data available: No

Additional Information
Explanation for why data not available: Data collected for this study are proprietary of the Hospital Authority of Hong Kong, which granted researchers permission and access to data. The data that support the findings of this study are available from this authority, but restrictions apply to the availability to these data (information on the cost is available from the corresponding author). The analytic codes are available from the corresponding author on request.
